# RNAi Screening Implicates a SKN-1–Dependent Transcriptional Response in Stress Resistance and Longevity Deriving from Translation Inhibition

**DOI:** 10.1371/journal.pgen.1001048

**Published:** 2010-08-05

**Authors:** Jinling Wang, Stacey Robida-Stubbs, Jennifer M. A. Tullet, Jean-François Rual, Marc Vidal, T. Keith Blackwell

**Affiliations:** 1 Joslin Diabetes Center, Harvard Stem Cell Institute, and Department of Pathology, Harvard Medical School, Boston, Massachusetts, United States of America; 2 Center for Cancer Systems Biology (CCSB) and Department of Cancer Biology, Dana-Farber Cancer Institute, Boston, Massachusetts, United States of America; 3 Department of Genetics, Harvard Medical School, Boston, Massachusetts, United States of America; Stanford University Medical Center, United States of America

## Abstract

*Caenorhabditis elegans* SKN-1 (ortholog of mammalian Nrf1/2/3) is critical for oxidative stress resistance and promotes longevity under reduced insulin/IGF-1–like signaling (IIS), dietary restriction (DR), and normal conditions. SKN-1 inducibly activates genes involved in detoxification, protein homeostasis, and other functions in response to stress. Here we used genome-scale RNA interference (RNAi) screening to identify mechanisms that prevent inappropriate SKN-1 target gene expression under non-stressed conditions. We identified 41 genes for which knockdown leads to activation of a SKN-1 target gene (*gcs-1*) through *skn-1*-dependent or other mechanisms. These genes correspond to multiple cellular processes, including mRNA translation. Inhibition of translation is known to increase longevity and stress resistance and may be important for DR–induced lifespan extension. One model postulates that these effects derive from reduced energy needs, but various observations suggest that specific longevity pathways are involved. Here we show that translation initiation factor RNAi robustly induces SKN-1 target gene transcription and confers *skn-1*-dependent oxidative stress resistance. The accompanying increases in longevity are mediated largely through the activities of SKN-1 and the transcription factor DAF-16 (FOXO), which is required for longevity that derives from reduced IIS. Our results indicate that the SKN-1 detoxification gene network monitors various metabolic and regulatory processes. Interference with one of these processes, translation initiation, leads to a transcriptional response whereby SKN-1 promotes stress resistance and functions together with DAF-16 to extend lifespan. This stress response may be beneficial for coping with situations that are associated with reduced protein synthesis.

## Author Summary

The nematode *C. elegans* has proven to be an invaluable organism for elucidating mechanisms that influence aging. Here we used genome-scale RNA interference screening in *C. elegans* to identify mechanisms that regulate a set of genes that defend against oxygen radicals and other stresses. These genes are activated by the SKN-1 protein, which promotes longevity. We found that many biological processes influence the regulation of SKN-1–dependent stress defenses. These processes include mRNA translation, the mechanism by which proteins are synthesized. Previous work showed that reductions in translation slow aging, an effect that may be important in conserved longevity pathways. One model postulates that this increased longevity derives from reduced energy requirements, but here we determined that SKN-1–dependent stress gene expression and oxidative stress resistance are increased dramatically when translation initiation is inhibited. This effect is accompanied by increased longevity that depends largely upon SKN-1 acting in concert with DAF-16, a gene regulator that is widely implicated in longevity. We conclude that reductions in translation result in a stress gene activation response that increases both stress resistance and lifespan and may help the organism cope with situations that are associated with decreased protein synthesis.

## Introduction

Small molecules that react with proteins, lipids, and nucleic acids can damage cells catastrophically. Oxidative stress refers to damage caused by reactive oxygen species (ROS), but other reactive molecules are produced during metabolism of endogenous (endobiotic) or exogenous (xenobiotic) compounds. Oxidative or xenobiotic stress is central to the pathogenesis of diabetes, atherosclerosis, cirrhosis, and many other syndromes, and has been implicated in aging [1]–[6]. Eukaryotic cells handle reactive compounds through a detoxification system in which lipophilic molecules are solubilized (Phase 1), and reactive species that include ROS and products of the Phase 1 system are inactivated (Phase 2) and may be transported out of the cell (Phase 3) [7]–[9].

Many Phase 2 detoxification genes are induced coordinately in response to oxidative or xenobiotic stress. This stress response is important in the liver and several other tissues in mammals, in which it is mediated by the Nrf1/2/3 (NF-E2-related factor) transcription factors [9], [10]. In the nematode *C. elegans*, this conserved stress response is mediated by the Nrf protein ortholog SKN-1 [11]. In the intestine, which is the major detoxification organ in *C. elegans*, SKN-1 accumulates in nuclei and activates target genes in response to various stresses [11], [12]. The relationship between SKN-1 and its targets is more complicated than a simple on/off stress response, however. Under non-stressed conditions SKN-1 up- or down-regulates a wide range of genes, including Phase 1, Phase 2, and Phase 3 detoxification, membrane, lysosomal, proteasomal, metabolic, and regulatory protein genes, many of which seem to be direct targets [12]. SKN-1 responds to stress by upregulating narrower sets of detoxification genes, and under certain conditions some SKN-1 target genes are activated by SKN-1-independent mechanisms [12]–[14]. It remains to be determined how cellular processes and regulatory inputs modulate expression of these overlapping groups of SKN-1-regulated genes.

*C. elegans* has been particularly advantageous for identifying mechanisms that influence aging. It was discovered in *C. elegans* that lifespan is increased by reductions in insulin/IGF-1-like signaling (IIS), a pathway that has since been implicated in longevity in *Drosophila*, mammals, and possibly humans [15], [16]. In *C. elegans*, this increased longevity requires the FOXO ortholog DAF-16, which is inhibited by IIS. SKN-1 is inhibited by IIS in parallel to DAF-16, contributes to the increases in lifespan and stress resistance that derive from reduced IIS, and promotes longevity under normal conditions [17]. While these activities involve SKN-1 expression in the intestine, SKN-1 is also found in the ASI chemosensory neurons, which sense food availability and influence metabolism [11]. SKN-1 expression in these neurons is required for lifespan to be increased by dietary restriction (DR), a condition that extends lifespan in essentially every species examined [18]. SKN-1 is not required for interference with mitochondrial function to extend lifespan, however, indicating that it is not essential in all longevity pathways [17].

In species as diverse as yeast and rodents, longevity is also increased when mRNA translation is inhibited [19]. It is particularly important to understand how this occurs, because reductions in translation are involved in conserved mechanisms that promote longevity. From yeast to mice, lifespan is increased by inhibition of the TOR (target of rapamycin) signaling pathway, which integrates growth and nutrient availability cues and promotes translation [19], [20]. TOR signaling activates the ribosomal S6 protein kinase (S6K), which upregulates translation, and inhibits eIF4E-binding protein (4E-BP), an inhibitor of cap-dependent translation. In *Drosophila*, reversing these effects is required for rapamycin treatment to extend lifespan, and increased 4E-BP activity is important for lifespan to be extended by DR, a pathway that may involve TOR signaling [20], [21]. Moreover, reduction of S6K activity increases lifespan in yeast, *C. elegans*, *Drosophila*, and mice [19], [22]–[25]. While lower levels of translation might promote longevity simply by decreasing the energy requirements of protein synthesis [26], recent evidence indicates that specific regulatory mechanisms are involved. In yeast and *Drosophila*, reductions in overall translation levels lead to preferential translation of beneficial genes [21], [27]. Furthermore, some *C. elegans* studies have reported that DAF-16 is needed for lifespan to be extended when translation initiation is inhibited by RNAi or mutation of general translation factors [23], [28], [29], although other analyses of initiation factors suggest that DAF-16 is not required [22], [26], [30], [31]. Given that DAF-16 and SKN-1 are inhibited in parallel by IIS, and cooperate to regulate some target genes [17], it is an intriguing question whether SKN-1 might act in parallel to DAF-16 to promote longevity in response to reduced translation initiation.

Here we have employed genome-scale RNAi screening in *C. elegans* to identify mechanisms that prevent inappropriate expression of SKN-1-dependent stress defense genes. We identified 41 genes for which knockdown robustly activated a SKN-1-responsive promoter in the intestine, in most cases dependent upon *skn-1*. These genes represented multiple cellular processes that are monitored by SKN-1-dependent stress defenses. As several of these genes are involved in mRNA translation and protein synthesis, we investigated the involvement of *skn-1* in the effects of inhibiting translation initiation. We found that inhibition of genes involved in two different steps in translation initiation induced a robust transcriptional stress response, resulting in increased oxidative stress resistance that required SKN-1 but not DAF-16. In contrast, the accompanying longevity increases were mediated largely by the combined action of DAF-16 and SKN-1, indicating that these transcription factors are each crucial for the beneficial effects of translation suppression.

## Results

### Identification of genes that prevent constitutive SKN-1 target activation

**Figure 1:**
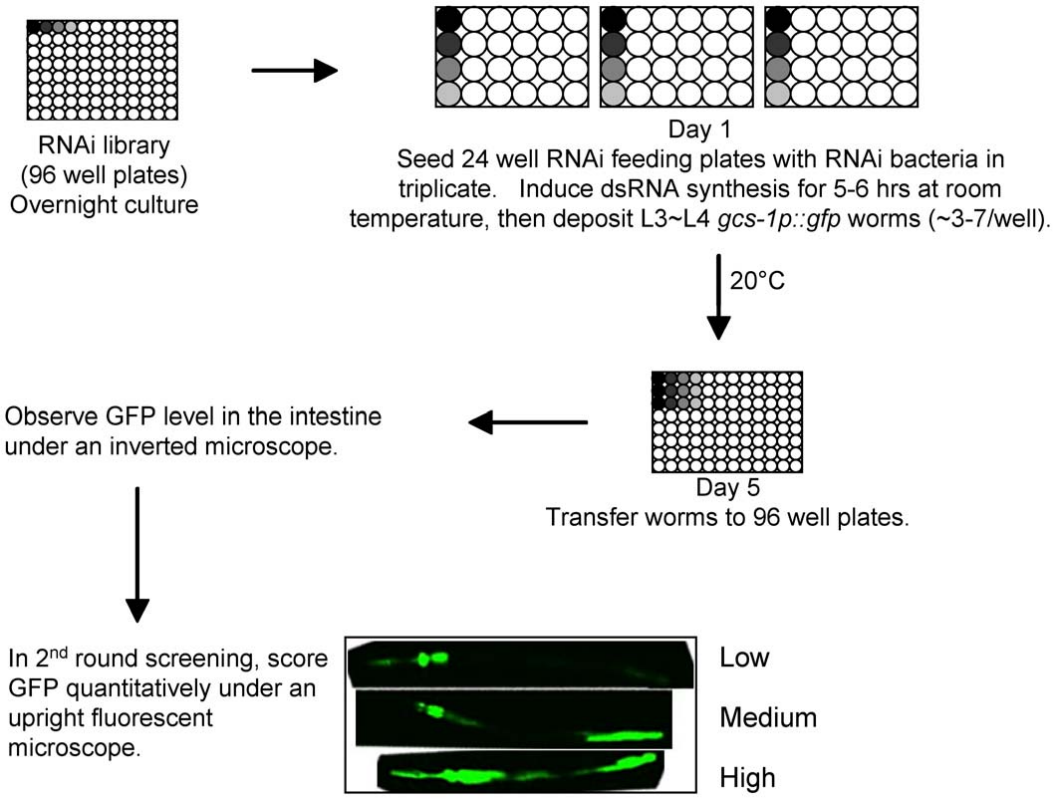
RNAi screening overview. After overnight culture, RNAi bacteria were seeded onto 24-well RNAi feeding plates in triplicate. dsRNA synthesis was induced for 5–6 hours, then 3–7 L3 or L4 *gcs-1p::GFP* worms were deposited into each well. After four days growth at 20°C, the triplicate worm samples were transferred to 96 well plates for assessment of the GFP signal in the intestine. Approximately 300 candidates were identified in the first screening round, in which a worm population was assessed rapidly as to whether the intestinal GFP signal was elevated. Those cDNA clones were analyzed in a quantitative second round of screening, in which intestinal GFP signal was scored as High, Medium, or Low as described [11], [17], [32] (Materials and Methods). Genes were scored as positive if *gcs-1* upregulation was robust in all three trials in the second round (Figure 2A; Table 1). Four distinct RNAi clones are represented by different shading in individual wells, with the remainder of the plates being arbitrarily left blank.

SKN-1 is inhibited from functioning constitutively in the intestine through phosphorylation by the IIS pathway kinases and glycogen synthase kinase-3 (GSK-3) [17], [32], but it is otherwise largely unknown how SKN-1 target genes are regulated. To identify mechanisms and cellular functions that limit expression of SKN-1 targets in the absence of stress, we used RNAi to screen for genes that prevent the Phase 2 gene *gcs-1* from being active constitutively in the intestine (Figure 1 and Figure S1A) [11]. *gcs-1* (γ-Glutamyl-Cysteine Synthetase heavy chain) is rate-limiting for glutathione (GSH) synthesis, and is induced by SKN-1/Nrf proteins in diverse eukaryotes. In the intestine *gcs-1* is expressed at low levels under normal conditions, and is upregulated dramatically by oxidative stress [11], [12]. This regulation can be visualized using a reporter in which the *gcs-1* promoter drives expression of the green fluorescent protein (GFP) gene (*gcs-1p::GFP;* Figure S1A).

**Figure 2:**
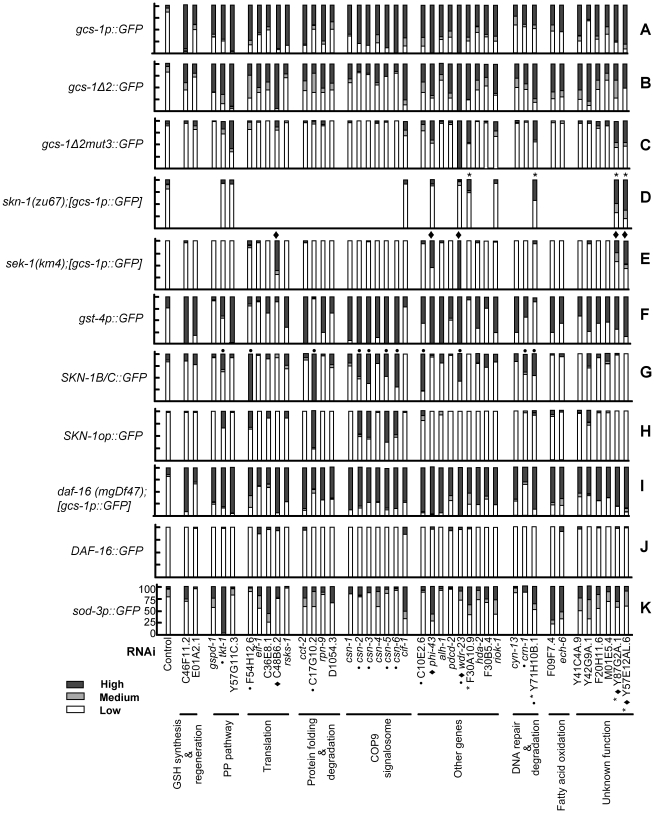
Analysis of genes that prevent constitutive *gcs-1* expression. Confirmed RNAi screening positives and additional COP9 signalosome subunits were examined by RNAi knockdown for effects on the indicated GFP reporters in L4 stage *C. elegans*. Reporters were scored for levels of nuclear GFP localization (SKN-1B/C::GFP, SKN-1op::GFP, DAF-16::GFP) or GFP expression in the intestine as High, Medium, or Low (Figure 1; Materials and Methods). Percentages of worms in each group were plotted on the Y axis in each panel. In each case a representative example of at least three RNAi experiments is shown (n>30 for each experiment). (A) *gcs-1p::GFP* expression. (B) Expression of the *gcs-1Δ2::GFP* reporter, which lacks a SKN-1-independent pharyngeal regulatory sequence and serves as a control for (C) (Figure S1A) [11]. (C) Expression of *gcs-1Δ2mut3::GFP*, in which an important SKN-1 binding site is mutated (Figure S1A) [11]. (D) *gcs-1p::GFP* expression in *skn-1(zu67)* mutants. Two independent transgenic lines each gave similar results. (E) *gcs-1p::GFP* expression in the *sek-1(km4)* mutant, in which stress-induced p38 signaling is blocked [41]. (F) Expression of the *gst-4p::GFP* promoter, a SKN-1 target [12], [40]. (G) Levels of nuclear SKN-1 expressed from *SKN-1B/C::GFP,* which encodes SKN-1 isoforms b and c (Figure S1B) [11]. (H) Nuclear accumulation of SKN-1 expressed from *SKN-1op::GFP,* which encodes all three SKN-1 isoforms (Figure S1B) [17]. (I) Expression of *gcs-1p::GFP* in *daf-16*(*mgDf47*) animals. (J) Presence of DAF-16::GFP (Table S6) in intestinal nuclei. (K) Activity of the DAF-16 target *sod-3* in the intestine. Black diamonds and asterisks indicate genes for which *gcs-1* was induced independently of *sek-1* or *skn-1*, respectively (summarized in Figure 3). Dots indicate genes that were associated with unambiguous accumulation of SKN-1::GFP in intestinal nuclei.

*C. elegans* is an advantageous organism for genome-scale RNAi screening, because RNAi can be performed in living animals by feeding [33]. We screened a *C. elegans* ORFeome library that consists of 11,511 full-length curated cDNA clones, or approximately 57% of the expressed genome (Figure 1) [34]. Two rounds of screening confirmed 37 “positive” genes for which RNAi resulted in robust and consistent expression of *gcs-1::GFP* in the intestine (Figure 1 and Figure 2A; Table 1). Our screen inevitably missed genes that are associated with developmental defects or modest RNAi-mediated *gcs-1* induction, such as *akt-1,* or *-2* [17]. However, it was reassuring that we identified two genes that are involved in GSH production (glutathione reductase: *C46F11.2* and the GCS regulatory subunit: *E01A2.1*, Table 1), because conditions that decrease GSH levels would be expected to upregulate *gcs-1* [11], [12]. We also identified *wdr-23*, which encodes an apparent ubiquitin ligase subunit that binds SKN-1 and may trigger its degradation [35]. Together, these last findings strongly support the validity of our screen.

**Table 1:** List of genes for which RNAi induced *gcs-1* expression in the intestine.

Functional group	Gene ID	Function (NCBI-KOGs description)
Glutathione regeneration	C46F11.2	mitochondrial glutathione reductase
Glutathione synthesis	E01A2.1	Glutamate-cysteine ligase regulatory subunit
Pentose phosphate pathway	B0035.5	*gspd-1*, Glucose-6-phosphate 1-dehydrogenase
Pentose phosphate pathway	F01G10.1	*tkt-1,*Transketolase
Pentose phosphate pathway	Y57G11C.3	6-phosphogluconolactonase - like protein
Fatty acid oxidation	F09F7.4	Enoyl-CoA hydratase
Fatty acid oxidation	T05G5.6	*ech-6*, Enoyl-CoA hydratase
Translation	F54H12.6	Elongation factor 1 beta/delta chain
Translation	T27F7.3b	*eif-1*, Translation initiation factor 1 (eIF-1/SUI1)
Translation	C36E8.1	RNA polymerase I transcription factor
Translation	C48B6.2	U3 small nucleolar ribonucleoprotein (snoRNP) component
Translation	Y47 D3A.16	*rsks-1*, Ribosomal protein S6 kinase
Protein folding & degradation	T21B10.7	*cct-2,* Chaperonin complex component
Protein folding & degradation	C17G10.2	Hsp90 co-chaperone CNS1 (contains TPR repeats)
Protein folding & degradation	T06D8.8	*rpn-9*, 26S proteasome regulatory complex
Protein folding & degradation	D1054.3	Suppressor of G2 allele of skp1
COP9 signalosome	Y59A8A.1	*csn-1*
COP9 signalosome	B0025.2	*csn-2*
COP9 signalosome	Y38C1AA.2	*csn-3*
COP9 signalosome	Y55F3AM.15	*csn-4*
COP9 signalosome	B0547.1	*csn-5*
COP9 signalosome	Y67H2A.6	*csn-6*
COP9 signalosome	K08F11.3	*cif-1*, COP9 Signalosome and eIF3 complex shared subunit
DNA repair & degradation	Y116A8C.34	*cyn-13*, Cyclophilin-type peptidyl-prolyl cis-trans isomerase
DNA repair & degradation	Y47G6A.8	*crn-1*, 5′-3′ exonuclease
DNA repair & degradation	Y71H10B.1	IMP-GMP specific 5′-nucleotidase
Other genes	C10E2.6	Monocarboxylate transporter
Other genes	K10C2.4	*phi-43,* Fumarylacetoacetase
Other genes	F54D8.3	*alh-1,* Aldehyde dehydrogenase
Other genes	R07E5.10	*pdcd-2*, mammalian Programmed Cell Death Protein homolog
Other genes	D2030.9	*wdr-23*, WD40 repeat-containing protein
Other genes	F30A10.9	Predicted nucleic-acid-binding protein, contains PIN domain
Other genes	C08B11.2	*hda-2*, Histone Deacetylase
Other genes	F30B5.4	Similar to oxidative stress-induced growth inhibitor 2 in H. sapiens
Other genes	M01B12.5	*riok-1*, Similar to serine/threonine kinase RIO1.
Unknown function	Y41C4A.9	Uncharacterized conserved protein
Unknown function	Y42G9A.1	Unknown function
Unknown function	F20H11.6	Unknown function
Unknown function	M01E5.4	Unknown function
Unknown function	Y87G2A.1	Unknown function
Unknown function	Y57E12AL.6	Unknown function

The RNAi screen identified 37 of these genes, and four were identified subsequently by virtue of their being COP9 signalosome subunits (*csn-2, csn-3, csn-6, cif-1*; see text).

Most of the genes we identified are conserved across metazoa (Table 1, not shown), suggesting that the screen is likely to have identified conserved mechanisms that affect Phase 2 gene expression. These genes correspond to a variety of biological processes, including metabolism, mRNA translation, lipid oxidation, DNA degradation and repair, transcription, and protein folding and degradation (Table 1). Three genes (*csn-1*, *csn-4*, and *csn-5*) encode subunits of the COP9 signalosome, a complex that regulates cullin-based ubiquitin ligases by removing the NEDD8 modification from cullins [36]–[38]. Knockdown of the four other *C. elegans* COP9 signalosome subunits [39], which were not present in our library, also resulted in robust *gcs-1* activation (Figure 2A; Table 1). This brought the total number of genes that we analyzed further to 41. Most of our positive genes also influenced expression of the SKN-1 target gene *gst-4* (Figure 2F) [12], [40], suggesting that they may broadly affect SKN-1-dependent stress defenses.

### Multiple pathways regulate *gcs-1* expression

**Figure 3:**
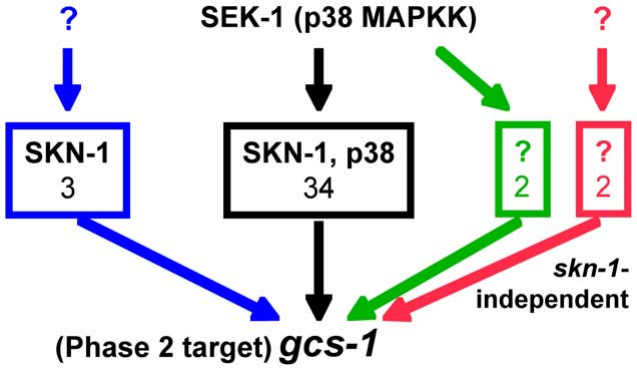
Pathways of *gcs-1* activation in the intestine. RNAi against 34 of the 41 genes we identified in this study resulted in *gcs-1* promoter activation through a canonical mechanism that required both *skn-1* and p38 MAPK signaling, as illustrated by requirement for the p38 MAPKK SEK-1 (black box)(Figure 2) [41]. In many of these cases the levels of SKN-1::GFP in intestinal nuclei were not dramatically increased (Figure 2), implying that *gcs-1* may be activated by SKN-1 through mechanisms besides increasing the overall levels of nuclear SKN-1 (see text). For three genes (*C48B6.2: snoRNP component*, *phi-43* and *wdr-23*; blue box) RNAi-induced *gcs-1* activation required SKN-1, but not p38 MAPK signaling (as revealed by *sek-1*-independence). For two genes (*F30A10.9: nucleic acid binding protein,* and *Y71H10B.1: IMP-GMP specific 5*′*-nucleotidase*), induction required SEK-1 but not SKN-1 (green box), implying that a different transcription factor was involved. In two cases (*Y87G2A.1* and *Y57E12AL.6*), *gcs-1* was activated independently of both SKN-1 and SEK-1 (red box).

We next investigated how the *gcs-1* promoter was activated by RNAi knockdown of the 41 genes identified in the screen. To determine whether SKN-1 was required for *gcs-1* induction, we first tested whether RNAi affected expression of a *gcs-1* promoter mutant that lacks an important SKN-1 binding site (*gcs-1Δ2mut3::GFP*)(Figure 2C and Figure S1A) [11]. If this mutated reporter was induced, we examined whether RNAi upregulated *gcs-1p::GFP* in a *skn-1* genetic mutant. For four genes we observed clear *skn-1* independent induction of the *gcs-1* promoter (*F30A10.9:* predicted nucleic acid binding protein, *Y71H10B.1:* IMP-GMP specific 5′-nucleotidase, *Y87G2A.1* and *Y57E12AL.6:* unknown function) (Figure 2D, marked with an asterisk, and Figure 3). Phosphorylation of SKN-1 in response to p38 stress-activated mitogen-activated kinase (MAPK) signaling is generally required for SKN-1 to accumulate in intestinal nuclei and activate target genes [17], [32], [41]. For most of our screening positives, *gcs-1* was not induced in animals that lack the MAPK kinase SEK-1, which is essential for p38 signaling [41] (Figure 2E). In contrast, and consistent with a recent study [35], *wdr-23* knockdown robustly activated *gcs-1p::GFP* in the *sek-1* null background (Figure 2E, marked with a diamond). This was also true for four other genes (*C48B6.2*: snoRNP component, *phi-43*: Fumarylacetoacetase, *Y87G2A.1* and *Y57E12AL.6*: unknown function). Knockdown of the last two genes also activated *gcs-1* independently of *skn-1*. Thus, although intestinal *gcs-1* expression is generally SKN-1-dependent, *gcs-1* can be induced through pathways that are independent of SKN-1, p38 signaling, or both mechanisms (Figure 3).

Our screen was designed to identify mechanisms that regulate SKN-1 itself, or might influence parallel processes that limit *gcs-1* expression. To test whether the genes we identified inhibit nuclear accumulation of SKN-1, we performed RNAi in two strains that carry transgenes in which SKN-1 isoforms are fused to GFP (Figure S1B). Interestingly, only a minority of the genes that regulated *gcs-1* through a *skn-1*-dependent mechanism clearly affected the levels of SKN-1 in intestinal nuclei, including multiple COP9 signalosome subunits, *tkt-1*, *F54H12.6*: eEF1, *C17G10.2*: HSP-90 co-chaperone, *C10E2.6*: Monocarboxylate Transporter, and *wdr-23* (Figure 2G and 2H, marked with a dot). Presumably other genes that regulate *gcs-1* in a *skn-1*-dependent manner act through a mechanism other than simply increasing nuclear SKN-1 levels. We also investigated whether our positives influence *daf-16-*dependent functions, because DAF-16 regulates many stress defense genes [15], [16]. For each gene, RNAi robustly activated the *gcs-1* reporter in a *daf-16* null mutant (Figure 2I), confirming our previous finding that *gcs-1* is expressed independently of *daf-16* [17]. We did not detect robust accumulation of DAF-16::GFP in intestinal nuclei, but for several genes we observed induction of the *skn-1*-independent DAF-16 target reporter *sod-3* (superoxide dismutase), suggesting that some DAF-16-dependent genes were affected (Figure 2J and 2K) [17], [42]. Some genes we identified thus appear to influence stress defense pathways that include targets of both SKN-1 and DAF-16.

### Many genes that inhibit *gcs-1* expression limit stress resistance

**Figure 4:**
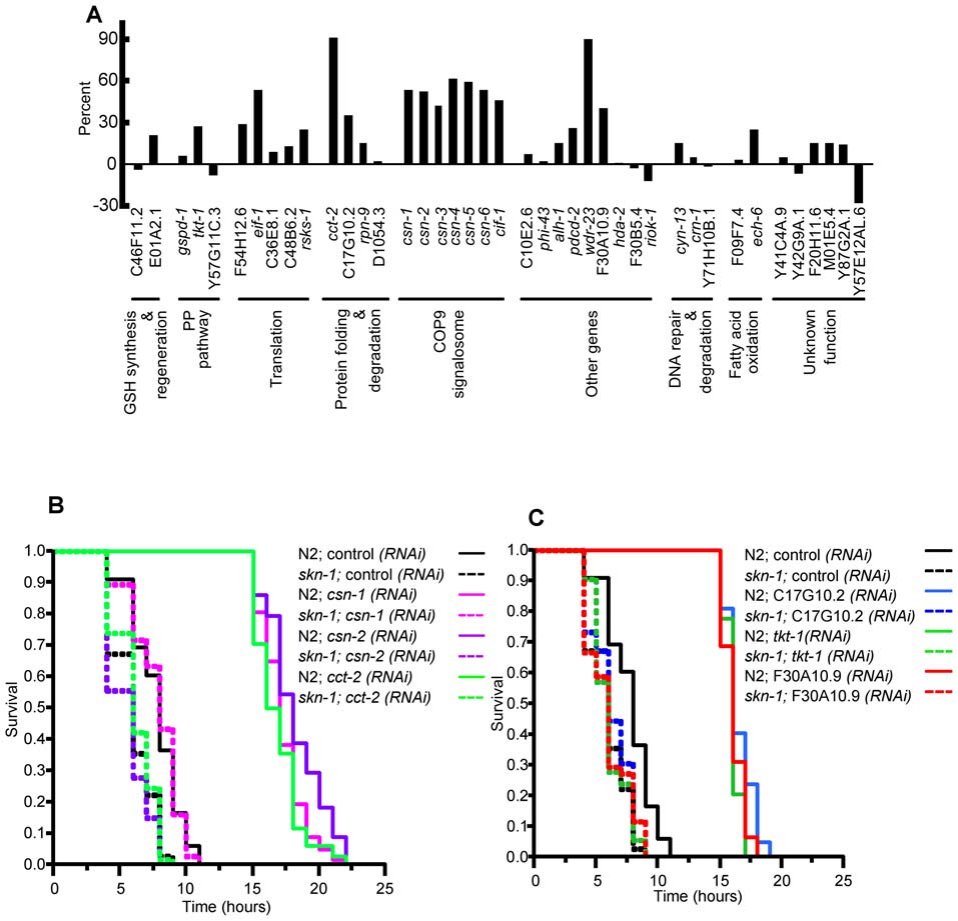
Effects of *gcs-1*-regulatory genes on stress resistance. (A) Effects on tert-butyl hydrogen peroxide (TBHP) resistance in wild-type (N2) animals. L4 worms were placed on RNAi or control bacteria for three days at 20°C, then transferred to assay plates containing a lawn of OP50 and 9.125 mM TBHP. Survival was then scored over a time-course. A bar graph shows the percent change in mean survival time for each gene compared to control RNAi. Representative experiments are shown here and plotted in Figure S2. Where only 2 experiments were performed, the experiment in which RNAi gave the less robust effect is graphed. Results of individual experiments, numbers of worms analyzed, and statistical analyses are presented in Table S1. (B, C) Effects on TBHP resistance in *skn-1(zu67*) animals. RNAi assays of selected genes were performed and analyzed as in (A), but using 15.4 mM TBHP. Representative experiments are presented as plots of proportional survival over time, with results and statistical analysis of individual experiments provided in Table S3. Note that in each case, the increase in stress resistance deriving from RNAi of these genes was almost completely dependent upon *skn-1*.

Our screen should identify genes for which RNAi activated the *gcs-1* promoter as a consequence of increased oxidative stress, but we also expected to identify regulatory genes and mechanisms that prevent *gcs-1* from being expressed constitutively. In the latter case, RNAi knockdown of these genes might increase oxidative stress resistance. Accordingly, for many of the genes we identified, RNAi dramatically enhanced resistance to treatment with the organoperoxide tert-butyl hydrogen peroxide (TBHP) (Figure 4A and Figure S2; Table S1). In addition to *wdr-23*, which has been implicated in stress resistance [35], robust effects were observed for many genes involved in translation, protein folding or degradation, and the COP9 signalosome. We observed comparable increases in TBHP resistance when a group of these genes was analyzed in a *daf-16* null mutant, indicating that *daf-16* is not required (Figure S2 and Figure S3; Table S2). We next asked whether a set of genes that had the greatest effects on stress resistance in N2 and *daf-16* animals could promote stress resistance in a *skn-1* mutant. In each case, RNAi largely failed to increase oxidative stress resistance when *skn-1* was lacking (Figure 4B and 4C; Table S3; see below). We conclude that many of the genes we identified are involved in mechanisms that limit oxidative stress resistance by modulating activity of SKN-1-dependent stress responses.

### Translation inhibition induces a transcriptional stress response that involves SKN-1

It was intriguing that five of our initial screening positives are involved in mRNA translation (Table 1), because several studies have reported that *C. elegans* lifespan and stress resistance are increased when genes that encode general translation factors or ribosomal proteins are inhibited by RNAi during adulthood [22], [23], [26], [28]–[31], [43]. Those longevity genes included two that we identified in our screen: the initiation factor eIF1 (*eif-1)* and the S6K ortholog *rsks-1* (Table 1). Our findings suggested that interference with mRNA translation might result in induction of SKN-1-dependent stress responses, and that SKN-1 might be involved in the stress resistance and lifespan extensions that derive from reduced translation. Accordingly, although our screen identified many interesting genes and candidate mechanisms that influence SKN-1-dependent stress responses, we directed our further efforts towards investigating the relationship between mRNA translation and SKN-1 function.

**Figure 5:**
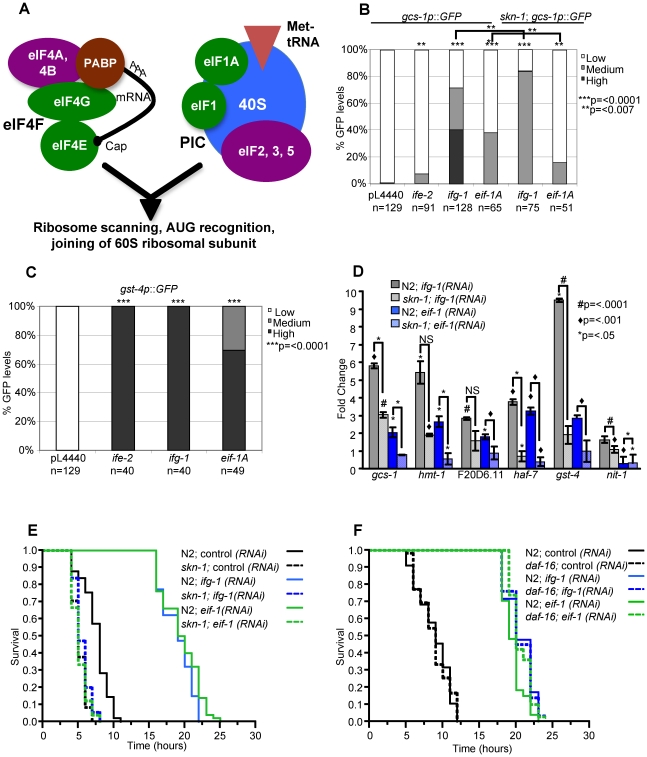
Induction of SKN-1–dependent target gene expression and stress resistance in response to translation initiation factor RNAi. (A) Translation initiation factors that were examined in this study. The eIF4F complex stabilizes capped mRNAs and activates them for translation by interacting with their 5′ cap and poly-A-binding protein (PABP) [44]. This interaction promotes binding of these mRNAs by the translation pre-initiation complex (PIC), which includes the 40 S ribosome subunit and the initiator tRNA. Subsequent steps in initiation follow this binding event. Initiation factors that we examined in this study are shown in green. (B) Activation of the *gcs-1p::GFP* reporter. N2 or *skn-1(zu67)* worms that carry the *gcs-1p::GFP* reporter were exposed to the indicated RNAi or control bacteria beginning at the L2 stage. They were scored for GFP fluorescence at day one of adulthood as in Figure 2, at which time the worms appeared normal and were laying eggs that hatched. p-values indicated above individual bars correspond to comparison with control RNAi. Similar reporter induction was observed after three days of initiation factor RNAi that began at adulthood day one, and no reporter activity was observed when control RNAi was performed in *skn-1(zu67)* animals (not shown). p values were calculated by the Chi^2^ method. (C) Activation of the *gst-4* reporter, scored as in (B). (D) Induction of endogenous SKN-1 target gene expression in response to translation initiation factor RNAi, analyzed by quantitative RT-PCR (qRT-PCR) performed in triplicate. RNAi was performed as in (B). Each gene assayed is upregulated under stress conditions [12]. A representative experiment is shown, in which Fold Change and p-values above individual bars refer to comparison to control RNAi. Additional qRT-PCR experiments and statistical analyses are described in Table S4. (E) Induction of *skn-1*-dependent stress resistance. After exposure to the indicated RNAi bacteria as in Figure 4, N2 or *skn-1(zu67)* worms were placed on plates containing 15.4 mM TBHP, then scored for survival over time. In each case, the worms appeared normal and were laying eggs when they were transferred to TBHP plates. In N2 but not *skn-1* mutant worms, stress resistance was dramatically enhanced by prior exposure to translation initiation factor RNAi. All experiments and statistics are provided in Table S3. (F) Comparison of TBHP resistance in N2 and *daf-16* mutant worms, performed and analyzed as in (E). *daf-16* was not required for the increases in oxidative resistance that derive from translation initiation factor RNAi.

We focused our analyses of translation on initiation factors because their lifespan phenotypes have been examined extensively, and because some studies indicated that their effects on lifespan involve DAF-16, which is inhibited by IIS in parallel to SKN-1 and may have some overlapping functions with SKN-1 (see Introduction). It is well established that mutation or adulthood RNAi knockdown of either eIF4G (IFG-1) or the somatically-expressed eIF4E isoform IFE-2 results in decreased protein synthesis, and increased lifespan and stress resistance [22], [23], [26], [31]. These longevity extensions appear to occur independently of any effects of translation inhibition on fecundity [23], [26]. Each of these factors is a subunit of the eIF4F complex, which circularizes and translationally activates mRNAs by linking their 5′ cap to poly-A-binding protein (Figure 5A) [44]. The eIF4F complex promotes binding of mRNA by the translation pre-initiation complex (PIC), which includes the 40S ribosomal subunit, a different set of initiation factors, and the methionyl tRNA that mediates initiation (Figure 5A) [44]. Here we have further examined stress and lifespan phenotypes associated with the eIF4F components IFE-2 and IFG-1, along with EIF-1 (Table 1) and EIF-1A *(H06H21.3)*. The latter two factors are components of the PIC, and are each involved in ribosome scanning and translation start codon selection [44]. Our experiments therefore investigated the effects of impairing two distinct mechanisms involved in translation initiation (Figure 5A).

We first investigated whether, in general, RNAi knockdown of translation initiation factors activates SKN-1-dependent stress responses. Initially we examined how initiation factor RNAi affected SKN-1 target gene promoter activity, as in the *eif-1*(PIC) analyses performed for our screen (Figure 2). Transcription from the transgenic *gcs-1* promoter was induced robustly by *ifg-1*(eIF4F) RNAi, and modestly by RNAi against *ife-2*(eIF4F) or *eif-1A*(PIC) (Figure 5B). This *gcs-1* induction was partially dependent upon *skn-1* (Figure 5B). In each case, translation factor RNAi also strongly activated the well-characterized SKN-1 target promoter *gst-4* (Figure 5C). We also analyzed effects on endogenous SKN-1-regulated gene expression, focusing on one factor each from eIF4F and the PIC (IFG-1 and EIF-1, respectively). We assayed mRNA production from two genes that are *skn-1*-dependent under both normal and oxidative stress conditions (*gst-4* and *nit-1*), along with other genes that are upregulated by SKN-1 in response to stress [12] (Figure 5D). Importantly, RNAi against either of these initiation factors dramatically increased expression of multiple endogenous SKN-1-regulated genes (Figure 5D; Table S4). When RNAi was performed in a *skn-1* mutant, this induction was much less robust or did not occur at all (Figure 5D; Table S4). We conclude that impairment of either of these two translation initiation complexes results in transcription-mediated stress responses in which SKN-1 plays a critical role.

We next examined how translation initiation factor RNAi affects oxidative stress resistance. RNAi against either *ifg-1*(eIF4F) or *eif-1*(PIC) dramatically increased TBHP resistance in either wild type or *daf-16* mutant animals (Figure 5E and 5F; Table S3). In contrast, these increases in stress resistance were essentially abolished in a *skn-1* mutant (Figure 5E and Table S3). Similar results were obtained in analyses of *ife-2*(eIF4F) and *eif-1A*(PIC) RNAi (Table S3). We conclude that the dramatic increase in SKN-1 target gene transcription that occurs after translation initiation factor RNAi results in oxidative stress resistance that depends upon *skn-1*, but not *daf-16*.

### Translation inhibition extends lifespan through *daf-16*- and *skn-1*–dependent mechanisms

**Figure 6:**
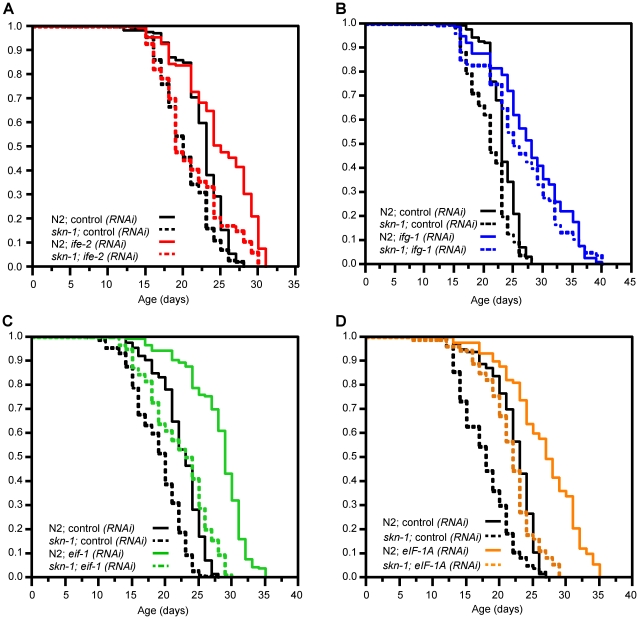
Importance of SKN-1 for lifespan extension deriving from translation initiation factor RNAi. (A) Survival plot showing effects of *ife-2*(eIF4F) RNAi. This lifespan extension was greatly reduced by the *skn-1(zu135)* mutation, which was used in all experiments in this figure. (B) Longevity extension by *ifg-1*(eIF4F) RNAi. Survival of *ifg-1(RNAi)* worms was not substantially decreased by *skn-1* mutation. (C,D) Longevity extension by *eif-1*(PIC) and *eif-1A*(PIC) RNAi. The longevity associated with inhibiting these genes was decreased but not eliminated by *skn-1* mutation. Note that overall survival of these RNAi animals is nevertheless significantly impaired in the *skn-1* background compared to N2. All longevity analyses were performed at 20°C, with lifespans measured from hatching and RNAi initiated at day 1 of adulthood. Each panel shows a composite of multiple experiments in which the populations shown were analyzed in parallel, with the proportion surviving indicated on the y-axis. These data are summarized in Table 2, with individual experiments described in Table S5.

To investigate whether *skn-1* contributes to the longevity increases that derive from RNAi knockdown of these translation initiation factors, we compared the effects of performing RNAi in the wild type strain N2, and two *skn-1* loss-of-function mutants (*skn-1(zu135) and skn-1(zu67)*). *ife-2*(eIF4F) RNAi did not consistently extend lifespan in these *skn-1* mutants, in contrast to results obtained in wild type animals (Figure 6A; Table 2 and Table S5). The mean lifespan associated with *ifg-1*(eIF4F) RNAi was only slightly reduced by lack of SKN-1, however (Figure 6B; Table 2 and Table S5). RNAi against either *eif-1*(PIC) or *eif-1A*(PIC) increased the mean lifespan of N2 worms to approximately the extent observed for *ifg-1*(eIF4F) RNAi (Figure 6C and 6D; Table 2 and Table S5). Lifespan was also increased when these last two genes were knocked down in *skn-1* mutants, but in each case the mean lifespan was markedly shorter than when the corresponding RNA was performed in N2 animals (Figure 6C and 6D; Table 2 and Table S5). In addition, the percent increase in mean lifespan associated with *eif-1*(PIC) RNAi was reduced in *skn-1(zu135)* mutants compared to N2 (12% vs. 26%, Table 2). The extent to which *skn-1* is required for RNAi-associated lifespan extension thus varies among these initiation factor genes.

**Table 2:** Lifespan analyses.

Strain	Mean Lifespan ± SEM 20° (days)	Median Lifespan	75^th^ Percentile 20°C (days)	p value (log-rank) against Control	% Lifespan Extension	N2	No. of Exp.	Figure
N2; control*(RNAi)*	22.64±0.2	23	25	-		264/320	3	6A
*skn-1(zu135);* control*(RNAi)*	20.29±0.2	20	23	-		348/382	3	6A
N2; *ife-2(RNAi)*	24.86±0.4	25	29	<.0001^a^	10	165/181	3	6A
*skn-1(zu135); ife-2(RNAi)*	21.11±0.4	19	24	<.0003^b^	4	153/173	3	6A
N2; control*(RNAi)*	22.30±0.2	23	25	-		225/271	4	6B
*skn-1(zu135);* control*(RNAi)*	21.02±0.2	21	23	-		236/260	3	6B
N2; *ifg-1(RNAi)*	27.91±0.5	28	33	<.0001^a^	25	186/242	4	6B
*skn-1(zu135); ifg-1(RNAi)*	26.21±0.5	25	32	<.0001^b^	25	175/204	3	6B
N2; control*(RNAi)*	22.43±0.2	23	25	-		280/296	5	6C
*skn-1(zu135);* control*(RNAi)*	19.87±0.3	20	22	-		211/220	4	6C
N2; *eif-1(RNAi)*	28.16±0.2	29	31	<.0001^a^	26	285/298	5	6C
*skn-1(zu135); eif-1(RNAi)*	22.18±0.3	23	26	<.0001^b^	12	207/210	4	6C
N2; control*(RNAi)*	22.28±0.3	23	25	-		120/122	2	6D
*skn-1(zu135);* control*(RNAi)*	17.94±0.4	18	21	-		97/100	2	6D
N2; *eif-1A(RNAi)*	26.84±0.5	27	31	<.0001^a^	20	130/140	2	6D
*skn-1(zu135); eif-1A(RNAi)*	21.62±0.4	22	24	<.0001^b^	21	106/107	2	6D
*skn-1(zu67);* control*(RNAi)*	18.33±0.4	17	22	-		111/122	2	N.A.
*skn-1(zu67); ife-2(RNAi)*	18.31±0.4	17	23	0.915^ c^	0	123/126	2	N.A.
*skn-1(zu67); ifg-1(RNAi)*	25.88±0.6	26	31	<.0001^ c^	41	101/111	2	N.A.
N2; control*(RNAi)*	21.40±0.3	21	23	-		99/100	2	N.A.
*skn-1(zu67);* control*(RNAi)*	17.68±0.3	17	21	-		209/221	4	N.A.
N2; *eif-1(RNAi)*	26.23±0.6	27	30	<.0001^a^	23	105/112	2	N.A.
*skn-1(zu67); eif-1(RNAi)*	20.42±0.4	19	24	<.0001^c^	15	183/201	4	N.A.
N2; control*(RNAi)*	22.51±0.3	22	24	-		65/70	1	7B
*daf-16(mgDf47);* control*(RNAi)*	19.74±0.4	20	23	-		113/116	2	7B
*daf-16(mgDf47); ife-2(RNAi)*	19.77±0.4	20	24	0.4388^d^	0	125/131	2	7B
N2; control*(RNAi)*	23.55±0.2	24	25	-		130/135	2	7C, 7E
*daf-16(mgDf47);* control*(RNAi)*	21.07±0.3	22	24	-		168/171	3	7C
*daf-16(mgDf47);skn-1(zu67);* control*(RNAi)*	16.91±0.3	17	19	-		141/160	3	7E
*daf-16(mgDf47); ifg-1(RNAi)*	23.61±0.4	25	28	<.0001^d^	12	165/168	3	7C
*daf-16(mgDf47); skn-1(zu67) ifg-1(RNAi)*	18.72±0.4	18	23	<.0001^e^	11	154/167	3	7E
N2; control*(RNAi)*	22.62±0.2	24	25	-		229/235	4	7D, 7F
*daf-16(mgDf47);* control*(RNAi*)	18.88±0.2	19	21	-		275/276	5	7D
*daf-16(mgDf47);skn-1(zu67);* control*(RNAi)*	16.16±0.2	16	19	-		194/206	4	7F
*daf-16(mgDf47); eif-1(RNAi)*	20.91±0.3	21	24	<.0001^d^	11	279/289	5	7D
*daf-16(mgDf47);skn-1(zu67); eif-1(RNAi)*	16.42±0.2	16	18	0.2232^e^	2	193/204	4	7F
N2; control*(RNAi)* – glc	24.40±0.2	25	26	-		122/126	2	7A
N2; control*(RNAi)* + glc	20.19±0.2	20	22	<.0001^f^	−17	178/178	3	7A
N2; *ife-2(RNAi)* + glc	19.96±0.2	20	22	0.2536^g^	−1	188/191	3	7A
N2; *ifg-1(RNAi)* + glc	19.49±0.2	19	22	0.2941^g^	−3	109/110	3	7A
N2; *eif-1*(RNAi) + glc	19.85±0.2	20	22	0.3771^g^	−2	176/176	3	7A

These combined results were derived from individual experiments that are described in Table S5. RNAi experiments are grouped and graphed in the indicated figures with controls that were performed in parallel. Lifespan extensions correspond to parallel control RNAi experiments. Numbers of animals are indicated as the total assayed (minus exclusions) over the total at the start of the experiment. p values refer to the following pL4440 RNAi controls: N2^a^, *skn-1(zu135)* ^b^, *skn-1(zu67)* ^c^, *daf-16(mgDf47)* ^d^, *daf-16(mgDf47); skn-1(zu67)* ^e^, N2-glc^f^, N2+glc^g^. glc means glucose.

**Figure 7:**
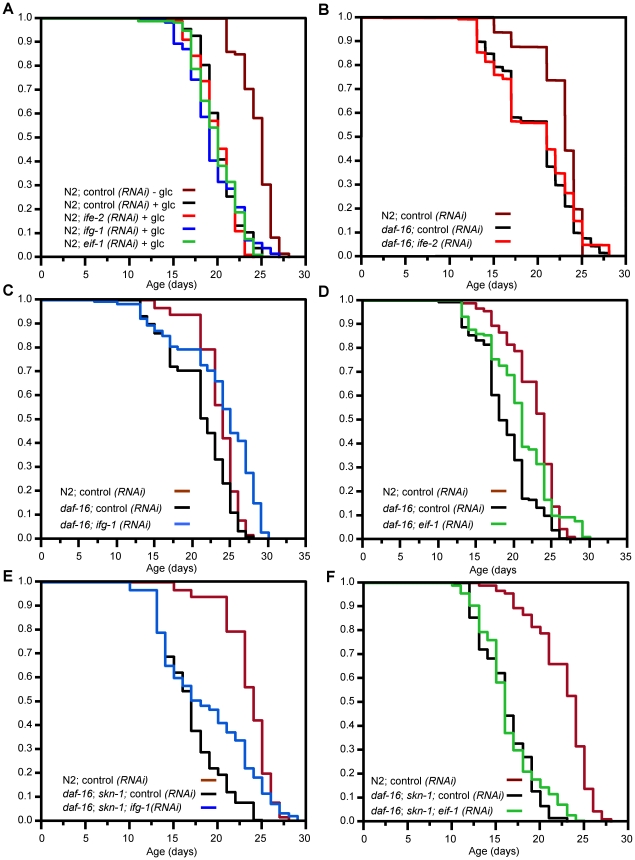
Lifespan extension in response to translation initiation factor RNAi is mediated primarily by DAF-16 and SKN-1. (A) Survival after exposure to translation initiation factor RNAi and 2% glucose. Glucose feeding increases IIS, which inhibits both DAF-16 and SKN-1 [17], [45]. Lifespan extension by translation factor inhibition is eliminated under these conditions. (B) *ife-2*(eIF4F) RNAi fails to extend lifespan in a *daf-16(mgDf47)* mutant. (C, D) Modest lifespan extension in response to *ifg-1*(eIF4F) or *eif-1*(PIC) RNAi in *daf-16(mgDf47)* animals. Note that survival of these RNAi animals is impaired in the *daf-16* background compared to N2 (Figure 6B and 6C). (E, F) Survival of *daf-16(mgDf47); skn-1(zu67)* double mutants exposed to *ifg-1*(eIF4F) or *eif-1*(PIC) RNAi. In this genetic background *ifg-1*(eIF4F) RNAi extends lifespan exclusively among the longest-lived worms, and *eif-1*(PIC) RNAi has only a negligible effect. Experiments were performed and plotted as in Figure 6 and are summarized in Table 2, with individual experiments described in Table S5.

As noted in the Introduction, results among laboratories differ with respect to whether *daf-16* is needed for the lifespan extensions associated with translation initiation factor RNAi. While these apparent discrepancies might derive simply from differences in experimental conditions, this is an important question to explore further. If *daf-16* is not involved in these effects, for example, it would suggest that translational suppression affects lifespan through novel mechanisms that do not intersect with the IIS pathway [26]. Our finding that SKN-1 contributes to these lifespan extensions in some cases suggests that those increases might involve DAF-16 and SKN-1 acting together. To test this idea, we first investigated whether these lifespan extensions occur under conditions of glucose feeding. In *C. elegans* glucose feeding inhibits DAF-16 by increasing IIS pathway activity, and thereby largely prevents mutations that reduce IIS from extending *C. elegans* lifespan [45], [46]. Given that IIS inhibits both DAF-16 and SKN-1 [17], glucose feeding should also reduce SKN-1 function in parallel to DAF-16, and therefore should block the pro-longevity effects of translation initiation factor RNAi if these two transcription factors are required. Consistent with published findings [46], we found that glucose feeding shortened the lifespan of N2 worms that were fed control RNAi (Figure 7A; Table 2). Importantly, the mean lifespans of *ife-2(RNAi)*(eIF4F), *ifg-1(RNAi)*(eIF4F), and *eif-1(RNAi)*(PIC) animals were even more dramatically decreased by glucose feeding, which prevented RNAi from extending lifespan in each case (Figure 7A; Table 2). This last finding suggests that the longevity benefits of inhibiting translation initiation are abrogated by upregulation of IIS signaling, supporting the idea that they may be largely dependent upon SKN-1 and DAF-16.

To test the above model directly, we investigated how the lifespan extensions associated with RNAi against *ife-2*(eIF4F), *ifg-1*(eIF4F), and *eif-1*(PIC) are affected by mutation of *skn-1* and *daf-16*, either individually or simultaneously. Under our conditions the prolongevity effect of *ife-2*(eIF4F) RNAi was essentially prevented by mutation of *daf-16* (Figure 7B; Table 2), consistent with a previous report [23]. Knockdown of either *ifg-1*(eIF4F) or *eif-1*(PIC) extended the mean lifespan of a *daf-16* mutant, but to a lesser extent than was characteristic of N2 animals (Figure 7C and 7D; Table 2). This result is largely consistent with previous evidence that the *ifg-1(RNAi)* lifespan extension requires *daf-16* [23], [28]. A homozygous *daf-16; skn-1* double mutant develops into adults that appear normal, but are characterized by a slightly reduced lifespan compared to either single mutant allele (Table 2). In this double mutant *ifg-1*(eIF4F) RNAi resulted in a modest lifespan increase that was confined largely to the longest-lived animals (compare median and 75%-ile lifespans; Figure 7E; Table 2). While mutation of *skn-1* on its own affected the mean lifespan of *ifg-1(RNAi)* animals only minimally (Figure 6B; Table 2), in the *daf-16* mutant concurrent loss of *skn-1* activity decreased *ifg-1(RNAi)* mean lifespan and altered the shape of the survival curve (compare results obtained in *daf-16* and *daf-16; skn-1* mutants; Figure 7C and 7F; Table 2). This suggests that lack of *daf-16* not only blunted the beneficial effects of *ifg-1*(eIF4F) RNAi, but also sensitized these animals to lack of *skn-1*. Importantly, *eif-1*(PIC) RNAi essentially failed to extend lifespan in the *daf-16; skn-1* double mutant (Figure 7F; Table 2). When our individual trials were combined, the average percent increase in lifespan associated with *eif-1*(PIC) RNAi in the *daf-16; skn-1* double mutant (2%) was significantly different from that seen in N2 (p = .0028, Student's t-test), *skn-1(zu67)*(p = .0937), or *daf-16(mgDf47)*(p = .0469)(Table 2 and S5). These analyses indicated that DAF-16 and SKN-1 each contributed significantly to the lifespan extension associated with *eif-1*(PIC) RNAi. Together, our findings provide strong support for the idea that DAF-16 is critical for the effects of translation initiation factor RNAi on lifespan, and additionally indicate that for some factors SKN-1 plays an important and possibly overlapping role.

## Discussion

### Various biological processes limit the activity of SKN-1/Nrf–dependent stress defenses

In this study we have used RNAi screening to identify mechanisms that prevent SKN-1-dependent stress response genes from being expressed inappropriately under normal conditions. The list of genes we detected is not complete, because we screened only about 60% of the expressed genome and employed stringent criteria for positive selection, and would have missed genes that prevented development. Nevertheless, our screen revealed that a variety of mechanisms and biological processes influence activity of SKN-1 and its target genes. For example, the proportion of these genes for which RNAi clearly increased the levels of SKN-1::GFP in intestinal nuclei was surprisingly low (about 25%, Figure 2). These particular positives could regulate SKN-1 itself, or processes that influence SKN-1 directly. Importantly, most of the remaining positives affected *gcs-1* activity in a *skn-1*-dependent manner, suggesting that they influence mechanisms that act on target genes in parallel to SKN-1, or might modify SKN-1 to increase its activity but not its concentration in the nucleus. We also identified genes that affect *gcs-1* expression independently of *skn-1*, p38 signaling, or both mechanisms (Figure 2 and Figure 3). In other species, considerable attention has been focused on regulation of the SKN-1 ortholog Nrf2 by the ubiquitin ligase and possible redox sensor Keap1, which targets Nrf2 for degradation in the absence of stress [9], [10], [47]. However, the related mammalian proteins Nrf1 and Nrf3 do not appear to be regulated by Keap1, a sequence ortholog of which seems to be lacking in *C. elegans*. Our results predict that Nrf proteins and their target genes, like SKN-1 and *gcs-1*, are likely to be regulated by a complex web of cellular processes and signaling pathways.

Many of the genes we identified are involved in metabolic processes (Table 1), which is not surprising given that SKN-1-regulated genes defend against stress deriving from excess levels of ROS or other reactive compounds [12]. For example, we identified several genes in the pentose phosphate pathway, which produces the critical reductant NADPH. Lack of PHI-43, which catalyzes the last step in tyrosine degradation, results in lethality that derives from accumulation of toxic tyrosine metabolites [48]. Similarly, monocarboxylate transporters (*C10E2.6*) prevent excessive accumulation of small molecules such as pyruvate, lactate, and ketone bodies. SKN-1 regulates numerous genes under normal conditions, and responds to stresses by inducing overlapping sets of stress defense genes [12]. Several of the genes we identified in this screen are themselves upregulated transcriptionally by SKN-1 (*C46L11.2*-glutathione reductase, *E01A2.1*-GCS regulatory subunit, *phi-43*, *alh-1*, *rpn-9*, Table 1) [12], suggesting that SKN-1 is involved in homeostatic feedback regulation of various cellular processes. It may be important to regulate SKN-1 target gene activity tightly for many reasons: metabolite levels profoundly influence metabolism, IIS and other signaling pathways are affected by redox conditions [49], and excessive GSH could upset protein folding by inhibiting disulfide bond formation [50].

Several of our screening positives are involved in protein folding or degradation, many of which affected SKN-1 nuclear accumulation. WDR-23 appears to target SKN-1 directly for degradation (Table 1) [35]. This would seem to provide a model for how SKN-1 could be affected by the COP9 signalosome, which sustains cullin activity [36]–[38]. However, p38 signaling is required for *gcs-1* to be upregulated by loss of COP9 signalosome genes, in contrast to *wdr-23*, suggesting that the COP9 signalosome regulates SKN-1 at a different step (Figure 2E) [35]. We observed particularly strong effects on SKN-1 nuclear accumulation after knockdown of an HSP-90 co-chaperone (*C17G10.2*)(Figure 2G and 2H), but knockdown of the chaperonin *cct-2* and the proteasome lid subunit *rpn-9* upregulated SKN-1 target genes in a *skn-1*-dependent manner without detectably increasing the presence of SKN-1 in nuclei (Figure 2). These various genes associated with protein homeostasis thus may influence SKN-1 target gene expression through multiple pathways. RNAi against a set of these genes increased oxidative stress resistance in a manner that was almost completely dependent upon *skn-1* (*csn-1, csn-2, cct-2, C17G10.2*; Figure 4B and 4C; Table S3). Previous studies have shown that SKN-1 upregulates many proteasomal and other genes involved in protein turnover, including *rpn-9* [12], and that knockdown of several other proteasome or chaperonin subunits results in *skn-1*-dependent *gst-4* induction, or SKN-1 nuclear accumulation [40]. Perhaps SKN-1 helps maintain the proteasome and other mechanisms that promote protein homeostasis. The SKN-1 ortholog Nrf1 is required for inducible upregulation of proteasome genes in mouse fibroblasts [51], suggesting that this might be a conserved function of SKN-1/Nrf proteins.

### SKN-1 mediates effects of translation inhibition on stress resistance and longevity

Having identified screening positives that are involved in mRNA translation or ribosome function, including two known longevity genes (*eif-1*(PIC) and *rsks-1*, Table 1) [22], [23], [31], we investigated whether SKN-1 contributes to the increases in stress resistance and lifespan that derive from inhibiting translation initiation. The dramatic increases in oxidative stress resistance that accompanied translation initiation factor RNAi did not require *daf-16* but were eliminated in a *skn-1* mutant (Figure 5E and 5F; Table S3), indicating that SKN-1 plays a critical role in the effects of translation inhibition on stress resistance. In contrast, the increases in lifespan that derive from inhibiting translation initiation seemed to depend largely upon the activity of both *daf-16* and *skn-1* (Table 2). DAF-16 on its own contributed to these increases for each gene that we analyzed (Table 2), consistent with several previous analyses of the effects of translation on aging (see Introduction). SKN-1 was less critical than DAF-16 for these longevity benefits, but nevertheless still played an important role. Most notably, SKN-1 contributed to the percent longevity increases associated with knockdown of *ife-2* and *eif-1*, and SKN-1 and DAF-16 together mediated the longevity increase that derived from *eif-1* knockdown, which was essentially eliminated in *daf-16; skn-1* double mutants (Table 2). Glucose feeding had a similar effect for each gene we examined, also consistent with DAF-16 and SKN-1 being important. Previous studies have disagreed with respect to the importance of *daf-16* for longevity deriving from inhibiting translation initiation (see Introduction), possibly because of differences in experimental conditions. By analyzing requirements for both DAF-16 and SKN-1, we have obtained strong support for the notion that these longevity benefits are mediated through effects on specific regulatory pathways, and not simply through reducing the consumption of resources by protein synthesis.

Notably, these effects of translation inhibition do not simply require these transcription factors to be present and functioning as they would under normal conditions, but also involve induction of stress response gene transcription. RNAi knockdown of each initiation factor we examined led to activation of SKN-1 target gene promoters (*ife-2*(eIF4F), *ifg-1*(eIF4F), *eif-1*(PIC), and *eif-1A*(PIC); Figure 2A, Figure 5B and 5C), and knockdown of either *ifg-1*(eIF4F) or *eif-1*(PIC) dramatically upregulated transcription of endogenous SKN-1 target genes (Figure 5D). These gene induction events were largely but not completely dependent upon *skn-1* (Figure 5B and 5D), suggesting that SKN-1 and additional stress defense regulators are involved. Consistent with this idea, knockdown of the initiation factor eIF2Bδ was reported to increase expression of a set of stress response genes, an effect that was partially dependent upon *daf-16* [29]. RNAi against translation initiation factors could potentially increase DAF-16 and SKN-1 activity simply by inhibiting IIS. However, SKN-1 accumulates in intestinal nuclei when IIS is decreased [17], and this did not occur after RNAi against the initiation factor genes we studied (Figure 2G and 2H; not shown). This suggests that translation initiation may not induce *skn-1*-dependent gene expression simply by reducing IIS or promoting nuclear accumulation of SKN-1, and instead may affect signaling or transcription pathways that function synergistically with SKN-1. Elucidation of these pathways may ultimately reveal why the requirements for *daf-16* and *skn-1* for longevity extension varied among the translation factors we examined (Table 2).

Several lines of evidence indicate that suppression of translation is important for the longevity extensions associated with reductions in TOR signaling, and possibly DR [19], [52]. In *Drosophila*, S6K downregulation and 4E-BP are required for *Drosophila* lifespan to be extended by treatment with rapamycin, which inhibits the TORC1 form of TOR kinase [20]. Furthermore, DR extension of *Drosophila* lifespan involves an increase in 4E-BP activity, which allows mitochondrial genes to be translated preferentially by virtue of their shorter 5′ untranslated regions [21]. If the latter mechanism is conserved in *C. elegans*, our results predict that reductions in translation would trigger this pathway in addition to the transcriptional effects we have described. We observed some remaining longevity extension associated with *ifg-1*(eIF4F) RNAi when both *skn-1* and *daf-16* were lacking, implying that an additional longevity-promoting mechanism was activated (Figure 7E; Table 2). In *C. elegans* the lifespan extensions associated with TOR inhibition require the PHA-4 transcription factor, but TOR, S6K, and ribosomal proteins appear to modulate lifespan independently of DAF-16, suggesting that multiple overlapping processes might be involved [22], [23], [53], [54]. One intriguing possibility is that the SKN-1-dependent transcriptional response we have observed here is induced as a consequence of particular genes being translated preferentially. It will be interesting to determine whether this transcriptional response is associated with other situations where translation is reduced, including DR.

Why would interference with translation initiation direct SKN-1 and DAF-16 to enhance stress resistance and longevity? Translation is reduced in response to nutrient deprivation, a condition under which it is presumably adaptive to mobilize mechanisms that promote stress resistance and survival. It might be beneficial to activate SKN-1-dependent antioxidant defenses simply *because* protein synthesis is reduced, since the highly reactive sulfur within methionine residues in cellular proteins may be an important protective antioxidant [55]. Alternatively, a reduction in protein synthesis might perturb metabolic processes so that reactive metabolites accumulate, making it helpful to increase the activity of small molecule detoxification mechanisms [12]. In addition, at least 30% of nascent polypeptides are normally degraded co-translationally by the proteasome because of inefficient folding or translation errors [56], [57]. If interference with translation initiation increased the fraction of polypeptides that were subject to degradation, upregulation of SKN-1 target genes involved in protein homeostasis could be adaptive. DAF-16-dependent processes are also likely to be beneficial for coping with translation perturbation, because when DAF-16 activity is very high *C. elegans* larvae enter a diapause state in which metabolic needs are sharply reduced, and stress resistance is elevated [58]. On the other hand, it may be advantageous to hold SKN-1- and DAF-16-dependent oxidative stress defenses in check under growth conditions, when IIS and translation rates are higher, because phosphatases that inhibit IIS are themselves inhibited by oxidation [49]. Irrespective of the biological rationale, our results show that interference with translation initiation triggers mechanisms that stimulate SKN-1-dependent transcription of stress defense genes, making it of considerable importance to identify those mechanisms.

## Materials and Methods

### *C. elegans* strains

Unless otherwise indicated, worms were cultured at 20°C on NGM plates that were seeded with a lawn of *E. coli* strain OP50-1 (*Caenorhabditis* genetics center). The *C. elegans* strains used are described in Table S6. The *Ex003[gcs-1p::GFP]* transgenic array expresses GFP driven by the *gcs-1* promoter (Figure S1) [11]. Strains in which this array was integrated into the genome were generated by UV treatment using a Stratalinker 2400 (Stratagene) set at 400 (×100 µJoules). Three independent *gcs-1p::GFP* integrated lines were generated, two of which were crossed into *skn-1(zu67)* to create strains LD1173 and LD1175. The extrachromosomal *Ex003[gcs-1p::gfp]* array was introduced into *daf-16* and *sek-1* mutant backgrounds by crossing. The following mutants were used:

#### *skn-1(zu135)*

A nonsense mutation that would prevent DNA binding by all three SKN-1 isoforms [59].

#### *skn-1(zu67)*

A nonsense mutation that lies within the coding regions of SKN-1a and SKN-1c but not SKN-1b (Figure S1), but is associated with all known *skn-1* phenotypes [11], [17], [18], [59].

#### *daf-16(mgDf47)*

A deficiency that removes *daf-16* coding regions [60].

#### *sek-1(km4)*

A putative null mutation that removes most of the coding region [41].

### Genome-scale RNAi screening

The *C.elegans* orfeome RNAi library that was screened consists of 11,511 distinct genes [34]. Screening RNAi was performed in 24 well plates in which NGM agar was supplemented with 50 µg/mL carbenicillin and 2 mM IPTG (Isopropyl β-D-1-thiogalactopyranoside)(Figure 1). Unseeded plates were stored in the cold room for < one week. RNAi bacteria were expanded in 96-well flat bottom blocks (QIAGEN, Valencia, CA) overnight at 37°C in 600 µL LB with 50 µg/mL carbenicillin. After seeding of individual bacterial clones, the RNAi plates were dried in a laminar flow hood and left at room temperature for 5–6 hours to induce dsRNA synthesis, then L3 or early L4 stage *gcs-1p::GFP* worms were deposited into each well (Day 1). After incubation for 4 days at 20°C (Day 5), worms were washed off with M9 containing 6 mM sodium azide (for immobilization), then transferred to 96-well black clear bottom plates (Corning) for observation under an inverted fluorescent microscope. This low azide concentration did not affect *gcs-1p::GFP* expression (not shown). For this 1^st^ round screen, RNAi for each gene was performed in triplicate. Clones were scored as positive if *gcs-1p::GFP* upregulation was unambiguously observed in at least one of the triplicate wells.

Approximately 300 candidate positives that were identified in the first round were examined for *gcs-1* reporter induction in a 2^nd^ screen in which (i) feeding RNAi was performed in 6-well plates, (ii) mothers were removed on day 3 by picking, and (iii) *gcs-1p::GFP* reporter expression in their progeny was scored on day 5 using an upright fluorescence microscope. Worms were transferred to a 2% agarose pad on a slide in M9, and covered with a glass slip prior to scoring. To discriminate intestinal autofluorescence from GFP, a triple band emission filter set (Chroma 6100) was used in conjunction with a narrow-band excitation filter (484/14 nm) [11]. Worms were scored for High, Medium, and Low *gcs-1p::GFP* expression as described below (Figure 1). At least 3 analyses of more than 30 worms each were performed for each RNAi clone. Positive genes for which robust *gcs-1* reporter activation was observed in all RNAi replicates were confirmed by sequencing. Additional COP9 signalosome subunit genes (*csn-2, -3, -6* and *cif-1*) were not present in the screening library but were subcloned from a later ORFeome version by standard Gateway reactions.

### RNAi

Unless otherwise indicated, feeding RNAi was carried out essentially as described, with HT115 carrying the empty pL4440 vector used as the control [61]. RNAi clones were grown with 12.5 µg/ml tetracycline and 100 µg/ml ampicillin. On the following day, cultures were diluted and grown to OD_600_ of 1 and induced with 0.6 mM IPTG. This culture was used to seed plates containing tetracycline, ampicillin and 0.6 mM IPTG.

### GFP reporter scoring

Essentially the same published scoring procedure was used to score intestinal GFP fluorescence for *gcs-1p::GFP* and other reporters (Figure 1) [11], [17], [32]. For promoter reporters, “High” indicates that GFP signal was detected at high levels throughout most of the intestine, while “Medium” refers to animals in which robust GFP signal was present only anteriorly or posteriorly. For the SKN-1::GFP fusion reporters, High indicated that a strong SKN-1:: GFP signal was present in all intestinal nuclei, and Medium that nuclear SKN-1:: GFP was present at high levels anteriorly, posteriorly or both, but barely visible midway through the intestine, or that a weak signal was observed in all intestinal nuclei. In general, L2 stage animals were placed on RNAi plates and allowed to develop to the L4 or early adult stage prior to scoring. p values were determined from a Chi^2^ test.

### RNA isolation and quantitative PCR

L2 stage larvae were fed RNAi or control bacteria until day 1 of adulthood. Animals were picked onto clean plates to minimize contamination, then total RNA was extracted from approximately 60 animals suspended in 25 µl of M9. RNA was extracted using Trizol (Sigma), and cDNA was synthesized using the Superscript Reverse Transcription Kit (Life Technologies). qRT-PCR was performed on an ABI 7700 instrument using the SYBER Green Real Time PCR kit (Life Technologies), the comparative Ct method, and normalization to *act-1*.

### Stress resistance assays

For TBHP resistance, L4 stage worms were fed with RNAi or control bacteria for three days at 20°C, then transferred to NGM plates that contained either 9.125 mM or 15.4 mM TBHP (Sigma) and were seeded with *E.coli* OP50. These plates were prepared two hours before transferring worms by adding TBHP (Sigma) to molten agar at 50–55°C. Each plate contained 20 worms, and the assay was performed in triplicate at 20°C. Worms were scored as dead when they did not respond to repeated gentle prodding with a platinum wire pick. All data were analyzed using JMP software.

### Lifespan analysis

Animals were maintained for at least two generations to assure health prior to analysis. Hermaphrodites were synchronized by timed egg laying for 8 hours and allowed to develop at 16°C on control RNAi. At day 1 of adulthood they were transferred to NGM plates containing 100 µg/ml FuDR and either RNAi or control pL4440 bacteria, with which they were fed throughout life. Lifespan assays were carried out at 20°C, with animals scored as dead or alive daily by gentle prodding with a pick. For glucose feeding, 2% glucose was included in the agar. Animals that crawled off the plate, ruptured, or died from internal hatching of progeny were excluded from analysis. Lifespans were measured from hatching. Survival plots, p values (Log-Rank), and proportional hazards were determined using JMP software.

## Supporting Information

Figure S1 (A) Diagram of the *gcs-1* promoter transgenes used in this study, which were described previously in [11]. The *gcs-1*Δ2 promoter lacks a region that confers *skn-1*-independent pharyngeal expression. An SKN-1 binding site that is required for most SKN-1-dependent promoter activity is mutated in the *gcs-1(Δ2mut3)::GFP* transgene. (B) SKN-1 isoforms (Wormbase). The three SKN-1 isoforms (SKN-1a (623aa), b (310aa) and c (533aa)) all share the same C-terminus, to which GFP has been attached. SKN-1b and SKN-1c are expressed from the *SKN-1B/C::GFP* transgene, which rescues all known *skn-1* phenotypes [11], [18], and all three isoforms are expressed from *SKN-1op::GFP*, which includes upstream operon sequences that drive SKN-1a expression [17].
(0.15 MB TIF)

Figure S2 Survival plots of representative TBHP resistance assays involving wild type *N2* and *daf-16*(*mgDf47*) worms, performed as described in Figure 4A. Data were analyzed by JMP and plotted with EXCEL. Statistical analyses are shown in Table S2.
(0.60 MB TIF).

Figure S3 TBHP resistance deriving from translation initiation factor RNAi is *daf-16*-independent. A survival assay that was performed and analyzed as in Figure 4A. Percent increase in mean survival compared to control is graphed. Representative experiments are shown here and plotted in Figure S2. All experiments and statistics are provided in Table S2. When analyzed side-by-side, N2 and *daf-16* worms were roughly comparable with respect to TBHP resistance (see Figure 5E and 5F; Table S3).
(0.18 MB TIF)

Table S1 Effects of RNAi clones on resistance of wild-type (*N2*) worms to TBHP. Individual experiments are listed that were performed as in Figure 4A. Representative survival plots are shown in Figure S2.
(0.13 MB DOC)

Table S2 Effects of RNAi clones on resistance of *daf-16* mutant worms to TBHP. Individual experiments are listed that were performed as in Figure 4A. Representative survival plots are shown in Figure S2.
(0.08 MB DOC)

Table S3 *skn-1*-dependence of TBHP resistance. Individual stress exposure experiments were performed as in Figure 4B and 4C. In each experiment, survival times were compared to pL4440 RNAi control. Note that the increases in stress resistance associated with translation initiation factor RNAi were consistently almost completely dependent upon *skn-1*, but did not require *daf-16*. Worms were censored if they bagged, escaped, or ruptured. p values were calculated by log-rank.
(0.08 MB DOC)

Table S4 qRT-PCR analyses of SKN-1 target gene expression. Analyses of endogenous SKN-1 target gene mRNA levels were performed as described in Figure 5D, and Materials and Methods. In each experiment, fold change refers to the relative RNA levels detected in RNAi-treated versus pL4440 control worms. Note that the extent of induction was generally decreased in *skn-1* mutants. Each value was obtained through a qRT-PCR analysis that was performed in triplicate. p values were calculated by Student's t test.
(0.08 MB DOC).

Table S5 Summary and statistical analysis of individual lifespan experiments. Data presented in Table 2, Figure 6, and Figure 7 were compiled from these experiments. In each case, RNAi treatment was performed in parallel with a pL4440 RNAi control sample, with the percent mean lifespan extension indicated. Worms were censored that bagged, escaped or ruptured. p values were calculated by log-rank.
(0.11 MB DOC).

Table S6 Strains used in this study, with references.
(0.04 MB DOC)
