# Supplementary figures and images for: RNAi Screening Implicates a SKN-1–Dependent Transcriptional Response in Stress Resistance and Longevity Deriving from Translation Inhibition
Source: PLoS Genet. 2010 Aug 5;6(8):e1001048. doi: 10.1371/journal.pgen.1001048 (PMC2916858; doi:10.1371/journal.pgen.1001048)

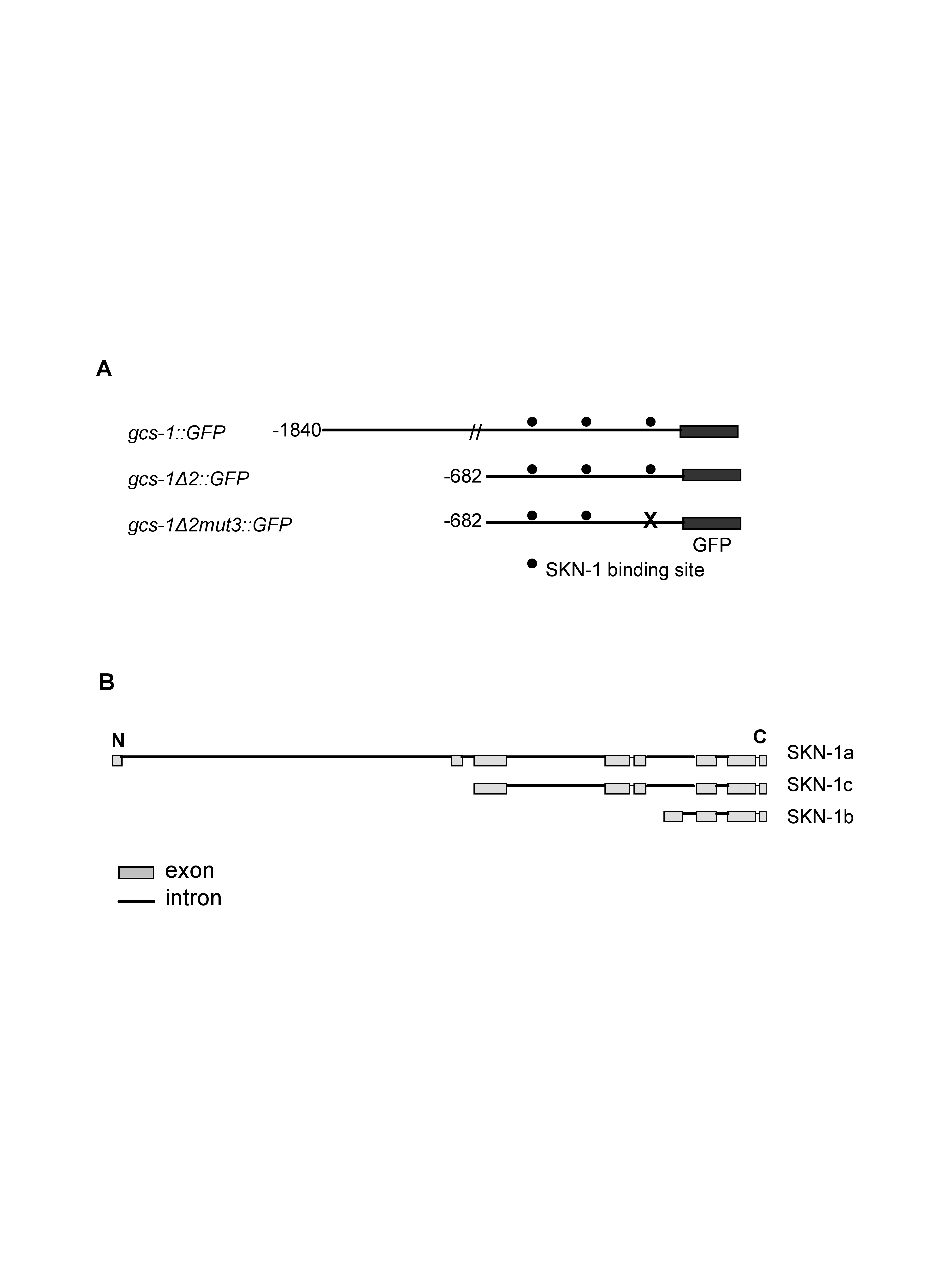

Supplement: Figure S1 — (A) Diagram of the gcs-1 promoter transgenes used in this study, which were described previously in [11]. The gcs-1Δ2 promoter lacks a region that confers skn-1-independent pharyngeal expression. An SKN-1 binding site that is required for most SKN-1-dependent promoter activity is mutated in the gcs-1(Δ2mut3)::GFP transgene. (B) SKN-1 isoforms (Wormbase). The three SKN-1 isoforms (SKN-1a (623aa), b (310aa) and c (533aa)) all share the same C-terminus, to which GFP has been attached. SKN-1b and SKN-1c are expressed from the SKN-1B/C::GFP transgene, which rescues all known skn-1 phenotypes [11], [18], and all three isoforms are expressed from SKN-1op::GFP, which includes upstream operon sequences that drive SKN-1a expression [17]. (0.15 MB TIF) [file pgen.1001048.s001.tif]

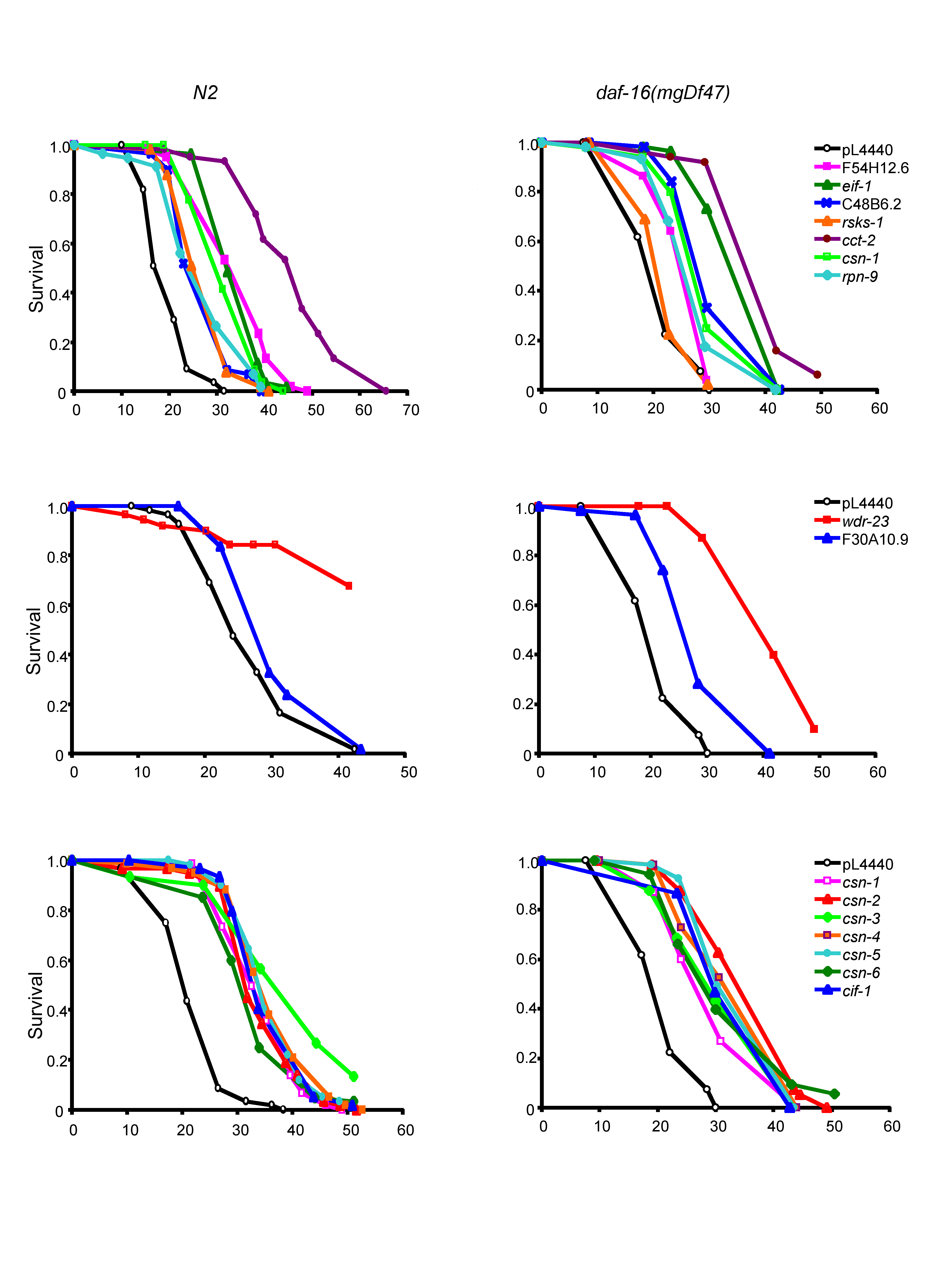

Supplement: Figure S2 — Survival plots of representative TBHP resistance assays involving wild type N2 and daf-16(mgDf47) worms, performed as described in Figure 4A. Data were analyzed by JMP and plotted with EXCEL. Statistical analyses are shown in Table S2. (0.60 MB TIF). [file pgen.1001048.s002.tif]

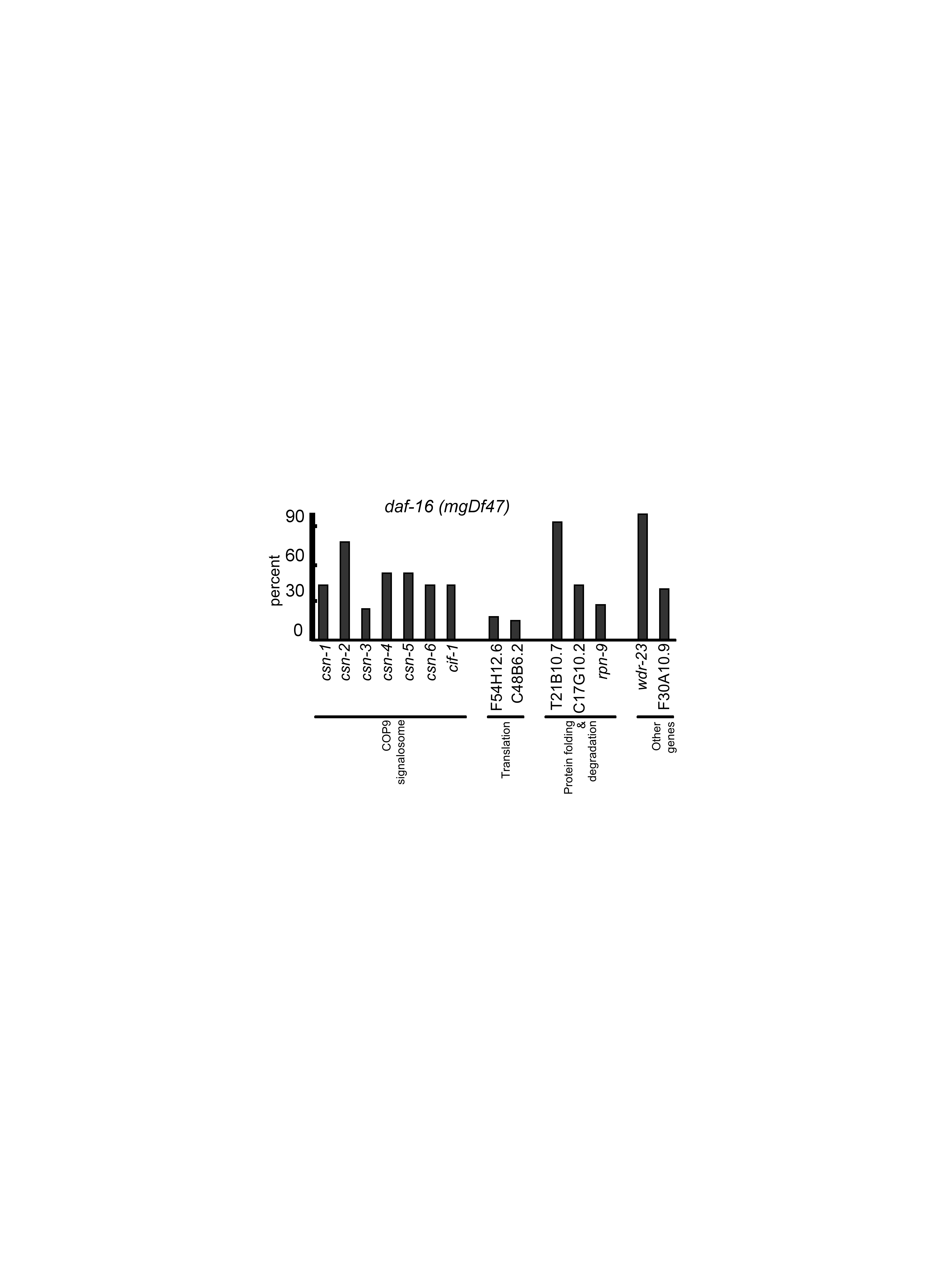

Supplement: Figure S3 — TBHP resistance deriving from translation initiation factor RNAi is daf-16-independent. A survival assay that was performed and analyzed as in Figure 4A. Percent increase in mean survival compared to control is graphed. Representative experiments are shown here and plotted in Figure S2. All experiments and statistics are provided in Table S2. When analyzed side-by-side, N2 and daf-16 worms were roughly comparable with respect to TBHP resistance (see Figure 5E and 5F; Table S3). (0.18 MB TIF) [file pgen.1001048.s003.tif]
