# Supplementary material for: RNAi Screening Implicates a SKN-1–Dependent Transcriptional Response in Stress Resistance and Longevity Deriving from Translation Inhibition
Source: PLoS Genet. 2010 Aug 5;6(8):e1001048. doi: 10.1371/journal.pgen.1001048 (PMC2916858; doi:10.1371/journal.pgen.1001048)
Supplement: Table S1 — Effects of RNAi clones on resistance of wild-type (N2) worms to TBHP. Individual experiments are listed that were performed as in Figure 4A. Representative survival plots are shown in Figure S2. (0.13 MB DOC) [file pgen.1001048.s004.doc]

Table S1. Effects of RNAi clones on resistance of wild-type (N2) worms to TBHP

| Functional group | RNAi  treatment | Mean survival time change % | Survival time change %  (75% worms are dead) | Worm number  n^a^ | *P*-value vs. control  (log-rank) |
| --- | --- | --- | --- | --- | --- |
|  |  |  |  |  |  |
| GSH regeneration | C46F11.2 | -3 | 1 | 58/2 | 0.7213 |
| GSH synthesis | E01A2.1 | 21 | 38 | 60/0 | 0.0011 |
|  |  |  |  |  |  |
| PP pathway | *gspd-1* | 6 | 14 | 57/3 | 0.5844 |
|  | *tkt-1* | 27 | 82 | 59/1 | 0.0039 |
|  | Y57G11C.3 | -18 | -22 | 58/2 | 0.0178 |
|  |  | -7 | -20 | 57/3 | 0.8628 |
|  |  |  |  |  |  |
| Fatty acid oxidation | F09F7.4 | 3 | 15 | 52/8 | 0.996 |
|  | *ech-6* | 25 | 20 | 59/1 | <0.0001 |
|  |  |  |  |  |  |
| Translation | F54H12.6 | 26 | 35 | 58/2 | <0.0001 |
|  |  | 29 | 3 | 60/0 | <0.0001 |
|  |  | 80 | 68 | 60/0 | <0.0001 |
|  | C36E8.1 | 9 | 1 | 55/3 | 0.0217 |
|  |  | 14 | 29 | 52/8 | 0.0012 |
|  | C48B6.2 | 13 | 13 | 57/3 | 0.0218 |
|  |  | 40 | 36 | 58/2 | <0.0001 |
|  | *rsks-1* | 25 | 28 | 51/9 | <0.0001 |
|  |  | 42 | 35 | 55/5 | <0.0001 |
|  |  |  |  |  |  |
| Protein folding & degradation | *cct-2* | 66 | 82 | 37/13 | <0.0001 |
|  |  | 91 | 66 | 60/0 | <0.0001 |
|  |  | 134 | 117 | 60/0 | <0.0001 |
|  | C17G10.2 | 35 | 35 | 58/2 | <0.0001 |
|  |  | 16 | -20 | 60/0 | <0.0001 |
|  |  | 64 | 60 | 58/2 | <0.0001 |
|  | *rpn-9* | 15 | 17 | 59/1 | <0.0001 |
|  |  | 40 | 59 | 56/3 | <0.0001 |
|  | D1054.3 | 2 | 2 | 59/1 | 0.9647 |
|  |  |  |  |  |  |
| COP9 signalosome | *csn-1* | 72 | 83 | 54/6 | <0.0001 |
|  |  | 53 | 50 | 59/1 | <0.0001 |
|  | *csn-2* | 46 | 23 | 55/5 | <0.0001 |
|  |  | 55 | 21 | 60/0 | <0.0001 |
|  |  | 52 | 46 | 58/2 | <0.0001 |
|  | *csn-3* | 13 | 0 | 59/1 | 0.0085 |
|  |  | 42 | 3 | 59/1 | <0.0001 |
|  |  | 79 | 130 | 52/8 | <0.0001 |
|  | *csn-4* | 104 | 120 | 60/1 | <0.0001 |
|  |  | 61 | 52 | 58/2 | <0.0001 |
|  | *csn-5* | 43 | 24 | 59/1 | <0.0001 |
|  |  | 75 | 36 | 57/3 | <0.0001 |
|  |  | 59 | 48 | 57/3 | <0.0001 |
|  | *csn-6* | 48 | 23 | 57/3 | <0.0001 |
|  |  | 57 | 21 | 59/1 | <0.0001 |
|  |  | 53 | 52 | 58/2 | <0.0001 |
|  | *cif-1* | 46 | 23 | 56/4 | <0.0001 |
|  |  | 34 | 4 | 60/0 | <0.0001 |
|  |  | 66 | 97 | 58/2 | <0.0001 |
|  |  |  |  |  |  |
| DNA repair & degradation | *cyn-13* | 17 | 1 | 56/4 | 0.0052 |
|  |  | 15 | 4 | 60/1 | 0.0002 |
|  | *crn-1* | 5 | 16 | 56/4 | 0.3268 |
|  | Y71H10B.1 | -1 | 1 | 60/0 | 0.5281 |
|  |  | 13 | 4 | 58/2 | 0.0006 |
|  |  |  |  |  |  |
| Other genes | C10E2.6 | 7 | 13 | 59/1 | 0.0486 |
|  | *phi-43* | 2 | 1 | 60/1 | 0.1700 |
|  |  | 5 | -20 | 58/2 | 0.0206 |
|  | *alh-1* | 15 | 15 | 56/4 | 0.0027 |
|  | *pdcd-2* | 26 | 20 | 59/1 | <0.0001 |
|  | *wdr-23* | 90 | 86 | 32/28 | <0.0001 |
|  |  | 162 | 126 | 17/43 | <0.0001 |
|  |  | 38 | na | 9--51 | 0.0003 |
|  | F30A10.9 | 40 | 86 | 59/1 | <0.0001 |
|  |  | 45 | 32 | 56/4 | <0.0001 |
|  |  | 17 | 3 | 54/6 | <0.0001 |
|  | *hda-2* | 0.8 | 2 | 52/8 | <0.0001 |
|  | F30B5.4 | -2 | 1 | 58/2 | 0.0361 |
|  | *riok-1* | -11 | -27 | 58/2 | 0.1997 |
|  |  | 12 | 4 | 60/0 | 0.0006 |
| Unknown functions |  |  |  |  |  |
|  | Y41C4A.9 | 5 | 13 | 57/3 | 0.2014 |
|  | Y42G9A.1 | -6 | 0 | 58/2 | 0.0005 |
|  | F20H11.6 | 15 | 21 | 58/2 | 0.0004 |
|  | M01E5.4 | 15 | 2 | 55/5 | 0.0109 |
|  |  | 40 | 3 | 60/0 | <0.0001 |
|  | Y87G2A.1 | 14 | 33 | 58/2 | 0.1889 |
|  | Y57E12AL.6 | -27 | -39 | 58/2 | <0.0001 |
|  |  | -4 | -20 | 54/6 | 0.7355 |
|  |  |  |  |  |  |
| control | pL4440 | 20.8 ± 0.8^b^ | 23.6^c^ | 54/6 |  |
|  |  | 25.1 ± 0.7^b^ | 25.5 ^c^ | 59/1 |  |
|  |  | 22.4 ± 0.5^b^ | 23.4 ^c^ | 56/4 |  |
|  |  | 32.3 ± 1.5^b^ | 42.7 ^c^ | 59/1 |  |
|  |  | 27.7 ± 1.1^b^ | 39.7 ^c^ | 58/2 |  |
|  |  | 22.7 ± 0.7^b^ | 26.5 ^c^ | 58/2 |  |
|  |  | 22.2 ± 0.7^b^ | 22.2 ^c^ | 54/6 |  |
|  |  | 19.9 ± 0.6^b^ | 23.7 ^c^ | 55/5 |  |
|  |  | 27.3 ± 1.1^b^ | 31.2 ^c^ | 54/6 |  |

(a) number of worms scored of dying of TBHP treatment/total number of worms censored (bagged, escaped or ruptured). In each experiment, survival times were compared to a pL4440 RNAi control. Mean + standard error (b) and 75% death (c) absolute survival times are indicated in hours for each individual control experiment.
