# Supplementary material for: RNAi Screening Implicates a SKN-1–Dependent Transcriptional Response in Stress Resistance and Longevity Deriving from Translation Inhibition
Source: PLoS Genet. 2010 Aug 5;6(8):e1001048. doi: 10.1371/journal.pgen.1001048 (PMC2916858; doi:10.1371/journal.pgen.1001048)
Supplement: Table S2 — Effects of RNAi clones on resistance of daf-16 mutant worms to TBHP. Individual experiments are listed that were performed as in Figure 4A. Representative survival plots are shown in Figure S2. (0.08 MB DOC) [file pgen.1001048.s005.doc]

Table S2. Effects of RNAi clones on resistance of *daf-16* mutant worms to TBHP

| Worm strain | Functional group | RNAi  treatment | Mean survival time change % | Survival time change %  (75% worms are dead) | Worm number  n^a^ | *P*-value vs. control  (log-rank) |
| --- | --- | --- | --- | --- | --- | --- |
|  |  |  |  |  |  |  |
| *daf-16(mgDf47)* | Translation | F54H12.6 | 6 | -2 | 37/23 | 0.6401 |
|  |  |  | 22 | 33 | 51/9 | <0.0001 |
|  |  |  | 19 | 30 | 56/4 | 0.0781 |
|  |  | C36E8.1 | -11 | 1 | 41/19 | 0.2106 |
|  |  |  | 5 | 5 | 58/2 | 0.0021 |
|  |  |  | 11 | 32 | 50/10 | 0.0049 |
|  |  | C48B6.2 | 10 | 9 | 51/9 | 0.0890 |
|  |  |  | 51 | 92 | 49/11 | <0.0001 |
|  |  |  | 14 | 32 | 47/13 | <0.0001 |
|  |  |  |  |  |  |  |
|  | Protein folding & degradation | T21B10.7 | 118 | 119 | 54/6 | <0.0001 |
|  |  |  | 93 | 90 | 48/5 | <0.0001 |
|  |  | C17G10.2 | 44 | 61 | 52/8 | <0.0001 |
|  |  |  | 42 | 33 | 49/3 | <0.0001 |
|  |  | rpn-9 | 27 | 27 | 54/6 | <0.0001 |
|  |  |  | 33 | 31 | 53/7 | <0.0001 |
|  |  |  |  |  |  |  |
|  | COP9 signalosome | csn-1 | 64 | 15 | 50/10 | <0.0001 |
|  |  |  | 43 | 99 | 51/9 | <0.0001 |
|  |  | csn-2 | 90 | 22 | 58/2 | <0.0001 |
|  |  |  | 76 | 96 | 56/4 | <0.0001 |
|  |  |  | 79 | 109 | 49/11 | <0.0001 |
|  |  | csn-3 | 3 | 7 | 53/7 | 0.0096 |
|  |  |  | 52 | 94 | 51/9 | <0.0001 |
|  |  |  | 25 | 73 | 49/11 | 0.0236 |
|  |  | csn-4 | 51 | -40 | 57/3 | <0.0001 |
|  |  |  | 65 | 99 | 51/9 | <0.0001 |
|  |  | csn-5 | 52 | -4 | 56/4 | <0.0001 |
|  |  |  | 67 | 96 | 51/9 | <0.0001 |
|  |  | csn-6 | 33 | 17 | 54/6 | <0.0001 |
|  |  |  | 54 | 94 | 50/10 | <0.0001 |
|  |  | cif-1 | 44 | 29 | 57/3 | <0.0001 |
|  |  |  | 62 | 93 | 60/0 | <0.0001 |
|  | Other genes | wdr-23 | 99 | 102 | 19/41 | <0.0001 |
|  |  |  | 99 | 122 | 43/17 | <0.0001 |
|  |  |  | 103 | 132 | 36/24 | <0.0001 |
|  |  | F30A10.9 | 39 | 37 | 49/11 | <0.0001 |
|  |  |  | 39 | 86 | 57/3 | <0.0001 |
|  |  |  | 50 | 74 | 55/5 | <0.0001 |
|  |  |  |  |  |  |  |
|  | control | pL4440 | 16.7 ± 0.4^b^ | 28.0^c^ | 59/1 |  |
|  |  |  | 18.9 ± 0.5 ^b^ | 22.3^c^ | 49/11 |  |
|  |  |  | 20.3 ± 1.0 ^b^ | 23.3^c^ | 49/11 |  |
|  |  |  | 17.8 ± 0.6 ^b^ | 21.4^c^ | 53/7 |  |
|  |  |  | 23.7 ± 1.0 ^b^ | 23.1^c^ | 51/9 |  |

(a) number of worms scored of dying of TBHP treatment/total number of worms censored (bagged, escaped or ruptured). In each experiment, survival times were compared to a pL4440 RNAi control. Mean + standard error (b) and 75% death (c) absolute survival times are indicated in hours for each individual control experiment.
