# Supplementary material for: RNAi Screening Implicates a SKN-1–Dependent Transcriptional Response in Stress Resistance and Longevity Deriving from Translation Inhibition
Source: PLoS Genet. 2010 Aug 5;6(8):e1001048. doi: 10.1371/journal.pgen.1001048 (PMC2916858; doi:10.1371/journal.pgen.1001048)
Supplement: Table S3 — skn-1-dependence of TBHP resistance. Individual stress exposure experiments were performed as in Figure 4B and 4C. In each experiment, survival times were compared to pL4440 RNAi control. Note that the increases in stress resistance associated with translation initiation factor RNAi were consistently almost completely dependent upon skn-1, but did not require daf-16. Worms were censored if they bagged, escaped, or ruptured. p values were calculated by log-rank. (0.08 MB DOC) [file pgen.1001048.s006.doc]

| Strain | RNAi  treatment | RNAi mean  Survival  (hours ± SEM) | 75^th^  Percentile (hours) | No.  RNAi animals | Control mean  Survival  (hours ± SEM) | 75^th^  Percentile  (hours) | No.  control animals | %  survival extension | p value  vs. control |
| --- | --- | --- | --- | --- | --- | --- | --- | --- | --- |
|  |  |  |  |  |  |  |  |  |  |
| N2 | *ife-2* | 20.57 ± 0.3 | 21 | 48/48 | 8.82 ± 0.3 | 11 | 57/57 | 130 | <.0001 |
|  | *ife-2* | 16.29 ± 0.2 | 17 | 34/34 | 8.84 ± 0.4 | 11 | 38/40 | 84 | <.0001 |
|  | *ifg-1* | 19.96 ± 0.3 | 20 | 76/76 | 8.92 ± 0.2 | 10 | 63/63 | 124 | <.0001 |
|  | *ifg-1* | 18.96 ± 0.3 | 21 | 53/53 | 7.49 ± 0.3 | 9 | 49/49 | 165 | <.0001 |
|  | *ifg-1* | 20.57 ± 0.3 | 21 | 46/46 | 8.82 ± 0.3 | 11 | 57/57 | 133 | <.0001 |
|  | *eif-1* | 19.48 ± 0.2 | 21 | 50/50 | 8.92 ± 0.2 | 10 | 63/63 | 118 | <.0001 |
|  | *eif-1* | 19.50 ± 0.4 | 22 | 50/50 | 7.49 ± 0.3 | 9 | 49/49 | 160 | <.0001 |
|  | *eif-1* | 19.56 ± 0.2 | 20 | 54/54 | 8.82 ± 0.3 | 11 | 57/57 | 122 | <.0001 |
|  | *eif-1A* | 19.17 ± 0.2 | 20 | 59/59 | 8.82 ± 0.3 | 11 | 57/57 | 117 | <.0001 |
|  | *csn-1* | 17.76 ± 1.4 | 26 | 49/52 | 8.33 ± 0.4 | 11 | 37/39 | 129 | <.0001 |
|  | *csn-1* | 17.19 ± 0.2 | 18 | 57/57 | 7.72 ± 0.2 | 9 | 79/83 | 123 | <.0001 |
|  | *csn-1* | 19.73 ± 0.3 | 21 | 44/44 | 8.84 ± 0.4 | 11 | 38/40 | 123 | <.0001 |
|  | *csn-2* | 22.29 ± 1.3 | 28 | 35/36 | 8.33 ± 0.4 | 11 | 37/39 | 168 | <.0001 |
|  | *csn-2* | 18.16 ± 0.3 | 20 | 44/44 | 7.72 ± 0.2 | 9 | 79/83 | 135 | <.0001 |
|  | *csn-2* | 15.92 ± 0.2 | 16 | 36/36 | 7.31 ± 0.2 | 8 | 55/55 | 117 | <.0001 |
|  | *cct-2* | 16.82 ± 0.3 | 18 | 34/34 | 7.72 ± 0.2 | 9 | 79/83 | 118 | <.0001 |
|  | C17G10.2 | 16.50 ± 0.2 | 17 | 42/44 | 7.72 ± 0.2 | 9 | 79/83 | 114 | <.0001 |
|  | C17G10.2 | 15.27 ± 0.2 | 15 | 49/49 | 7.31 ± 0.2 | 8 | 55/55 | 109 | <.0001 |
|  | F30A10.9 | 16.07 ± 0.1 | 17 | 58/61 | 7.72 ± 0.2 | 9 | 79/83 | 108 | <.0001 |
|  | *tkt-1* | 15.98 ± 0.1 | 16 | 54/54 | 7.72 ± 0.2 | 9 | 79/83 | 107 | <.0001 |
| *skn-1(zu67)* | *ife-2* | 7.49 ± 0.3 | 9 | 59/59 | 6.23 ± 0.3 | 6 | 43/43 | 20 | .0012 |
|  | *ifg-1* | 8.89 ± 0.2 | 10 | 61/61 | 7.56 ± 0.2 | 9 | 50/50 | 18 | <.0001 |
|  | *ifg-1* | 5.56 ± 0.1 | 6 | 55/55 | 5.16 ± 0.1 | 6 | 61/61 | 8 | .0347 |
|  | *eif-1* | 9.00 ± 0.2 | 10 | 60/60 | 7.56 ± 0.2 | 9 | 50/50 | 19 | <.0001 |
|  | *eif-1* | 5.17 ± 0.2 | 6 | 48/48 | 5.16 ± 0.1 | 6 | 61/61 | 0 | .8390 |
|  | *csn-1* | 9.12 ± 0.4 | 11 | 51/53 | 9.09 ± 0.3 | 11 | 43/43 | 0 | .1333 |
|  | *csn-1* | 7.76 ± 0.2 | 9 | 74/74 | 5.96 ± 0.2 | 7 | 67/67 | 30 | <.0001 |
|  | *csn-1* | 6.34 ± 0.4 | 8 | 35/35 | 6.23 ± 0.3 | 6 | 43/43 | -2 | .4844 |
|  | *csn-2* | 9.65 ± 0.3 | 11 | 54/54 | 9.09 ± 0.3 | 11 | 43/43 | 6 | .2406 |
|  | *csn-2* | 5.56 ± 0.2 | 7 | 54/54 | 5.96 ± 0.2 | 7 | 67/67 | -7 | .1937 |
|  | *csn-2* | 6.19 ± 0.2 | 8 | 51/51 | 5.66 ± 0.3 | 7 | 32/32 | 9 | .0959 |
|  | *cct-2* | 6.16 ± 0.2 | 7 | 57/57 | 5.96 ± 0.2 | 7 | 67/67 | 3 | .6323 |
|  | C17G10.2 | 6.27 ± 0.2 | 8 | 52/53 | 5.96 ± 0.2 | 7 | 67/67 | 5 | .1902 |
|  | C17G10.2 | 6.56 ± 0.3 | 8 | 45/45 | 5.66 ± 0.3 | 7 | 32/32 | 16 | 0.204 |
|  | F30A10.9 | 5.94 ± 0.3 | 8 | 51/51 | 5.96 ± 0.2 | 7 | 67/67 | 0 | .6156 |
|  | *tkt-1* | 6.06 ± 0.2 | 7 | 54/56 | 5.96 ± 0.2 | 7 | 67/67 | 2 | .9150 |
| *daf-16(mgDf47)* | *ife-2* | 19.45 ± 0.2 | 20 | 55/55 | 8.76 ± 0.3 | 11 | 66/66 | 122 | <.0001 |
|  | *ifg-1* | 21.98 ± 0.5 | 25 | 49/49 | 7.20 ± 0.2 | 8 | 50/50 | 205 | <.0001 |
|  | *ifg-1* | 20.57 ± 0.2 | 21 | 58/58 | 8.76 ± 0.3 | 11 | 66/66 | 135 | <.0001 |
|  | *eif-1* | 23.25 ± 0.4 | 25 | 60/60 | 7.20 ± 0.2 | 8 | 50/50 | 223 | <.0001 |
|  | *eif-1* | 20.65 ± 0.2 | 22 | 69/69 | 8.76 ± 0.3 | 11 | 66/66 | 136 | <.0001 |
|  | *eif-1A* | 19.86 ± 0.2 | 21 | 51/51 | 8.76 ± 0.3 | 11 | 66/66 | 127 | <.0001 |

Table S3. *skn-1*-dependence of TBHP resistance
