# Supplementary material for: RNAi Screening Implicates a SKN-1–Dependent Transcriptional Response in Stress Resistance and Longevity Deriving from Translation Inhibition
Source: PLoS Genet. 2010 Aug 5;6(8):e1001048. doi: 10.1371/journal.pgen.1001048 (PMC2916858; doi:10.1371/journal.pgen.1001048)
Supplement: Table S4 — qRT-PCR analyses of SKN-1 target gene expression. Analyses of endogenous SKN-1 target gene mRNA levels were performed as described in Figure 5D, and Materials and Methods. In each experiment, fold change refers to the relative RNA levels detected in RNAi-treated versus pL4440 control worms. Note that the extent of induction was generally decreased in skn-1 mutants. Each value was obtained through a qRT-PCR analysis that was performed in triplicate. p values were calculated by Student's t test. (0.08 MB DOC). [file pgen.1001048.s007.doc]

Table S4. qRT-PCR analysis of SKN-1 target gene expression

| Treatment | Gene | N2 *RNAi*  Fold Change | p value  vs.  N2 control (*RNAi*) | *skn-1(zu67)* *RNAi* Fold Change | p value  vs.  *skn-1(zu67)* control (*RNAi*) | Fold difference *skn-1*/N2 control (*RNAi*) | p value N2 gene (*RNAi*)  vs.  *skn-1(zu67)* gene (*RNAi*) |
| --- | --- | --- | --- | --- | --- | --- | --- |
|  |  |  |  |  |  |  |  |
| *ifg-1(RNAi)* | *gcs-1* | 5.79 | .0004 | 3.03 | <.0001 | 1.11 | .0088 |
|  | *gcs-1* | 6.64 | .0110 | 3.92 | .0003 | 0.57 | .0106 |
|  | *gcs-1* | 8.61 | .0015 | 0.25 | .0033 | 1.44 | .0017 |
|  | *hmt-1* | 5.41 | .0085 | 1.89 | .0016 | 1.83 | .0061 |
|  | *hmt-1* | 4.71 | .0485 | 3.66 | .0047 | 2.70 | .0314 |
|  | F20D6.11 | 2.83 | .0001 | 1.57 | .135 | 1.21 | .1452 |
|  | F20D6.11 | 1.31 | .0112 | 0.5 | .0001 | 2.31 | .0041 |
|  | F20D6.11 | 1.45 | .002 | 1.19 | .002 | 1.08 | .8420 |
|  | *haf-7* | 3.78 | .0005 | 0.70 | .0437 | 3.03 | .0124 |
|  | *haf-7* | .75 | .001 | .56 | .3284 | 2.66 | .0851 |
|  | *haf-7* | 2.03 | .0836 | 0.33 | .0048 | 0.44 | .0007 |
|  | *gst-4* | 9.50 | .009 | 1.90 | .0723 | 0.11 | .0001 |
|  | *gst-4* | 1.57 | .0798 | 0.71 | .3948 | 0.65 | .0031 |
|  | *nit-1* | 1.64 | .0960 | 1.07 | .0003 | 0.04 | .0001 |
|  | *nit-1* | 0.39 | .0142 | 0.25 | .0346 | 0.18 | .0202 |
|  |  |  |  |  |  |  |  |
| *eif-1(RNAi)* | *gcs-1* | 2.04 | .0197 | 0.77 | .0752 | 1.11 | .0015 |
|  | *gcs-1* | 0.82 | .011 | 0.40 | .0123 | 0.57 | .0140 |
|  | *gcs-1* | 3.80 | .0021 | 0.76 | .0045 | 1.44 | .0277 |
|  | *hmt-1* | 2.65 | .05 | 0.54 | .0153 | 1.83 | .0157 |
|  | *hmt-1* | 4.79 | .1797 | 0.44 | .0518 | 2.70 | .099 |
|  | *hmt-1* | 1.59 | .0170 | 0.74 | .7368 | .005 | .0001 |
|  | F20D6.11 | 1.79 | .0029 | .88 | .2447 | 1.21 | .001 |
|  | F20D6.11 | 1.20 | .07447 | 0.81 | .4789 | 2.31 | .1197 |
|  | F20D6.11 | 1.97 | .0200 | 0.99 | .8589 | 1.08 | .0949 |
|  | *haf-7* | 3.25 | .0011 | 0.38 | .0009 | 3.03 | .0013 |
|  | *haf-7* | 6.20 | .0105 | 1.33 | .3706 | 2.66 | .0062 |
|  | *haf-7* | 1.18 | .0327 | 0.96 | .0191 | 0.44 | .0515 |
|  | *gst-4* | 2.84 | .0938 | 0.97 | .9485 | 0.11 | .0001 |
|  | *gst-4* | 0.43 | .030 | 0.22 | .0409 | 0.03 | .0229 |
|  | *nit-1* | 0.27 | .0082 | 0.30 | .0522 | 0.04 | .0001 |
|  | *nit-1* | 0.64 | .0488 | 0.05 | .0076 | 0.18 | .0043 |
