# Supplementary material for: RNAi Screening Implicates a SKN-1–Dependent Transcriptional Response in Stress Resistance and Longevity Deriving from Translation Inhibition
Source: PLoS Genet. 2010 Aug 5;6(8):e1001048. doi: 10.1371/journal.pgen.1001048 (PMC2916858; doi:10.1371/journal.pgen.1001048)
Supplement: Table S5 — Summary and statistical analysis of individual lifespan experiments. Data presented in Table 2, Figure 6, and Figure 7 were compiled from these experiments. In each case, RNAi treatment was performed in parallel with a pL4440 RNAi control sample, with the percent mean lifespan extension indicated. Worms were censored that bagged, escaped or ruptured. p values were calculated by log-rank. (0.11 MB DOC). [file pgen.1001048.s008.doc]

Table S5. Summary and statistical analysis of individual lifespan experiments

| Strain | RNAi  treatment | Mean  RNAi  Lifespan  days ± SEM | 75^th^  Percentile  (days) | No.  of  RNAi animals | Mean  control  Lifespan  days ± SEM | 75^th^  Percentile  (days) | No.  of  control animals | %  lifespan extension | p value  vs. control |
| --- | --- | --- | --- | --- | --- | --- | --- | --- | --- |
| N2 | *ife-2* | 25.86 ± 0.5 | 29 | 49/55 | 22.94 ± 0.2 | 24 | 51/66 | 13 | <.0001 |
|  | *ife-2* | 25.83 ± 0.7 | 30 | 52/61 | 23.23 ± 0.6 | 26 | 55/61 | 11 | <.0001 |
|  | *ife-2* | 23.56 ± 0.6 | 29 | 66/67 | 21.58 ± 0.4 | 23 | 49/53 | 9 | .0006 |
|  | *ifg-1* | 27.80 ± 0.6 | 30 | 61/80 | 23.30 ± 0.3 | 26 | 70/81 | 19 | <.0001 |
|  | *ifg-1* | 22.70 ± 0.6 | 26 | 46/77 | 21.60 ± 0.4 | 22 | 39/59 | 5 | .001 |
|  | *ifg-1* | 28.99 ± 0.9 | 32 | 53/60 | 22.94 ± 0.2 | 24 | 51/66 | 26 | <.0001 |
|  | *ifg-1* | 35.00 ± 0.7 | 37 | 26/26 | 24.60 ± 0.3 | 26 | 65/65 | 42 | <.0001 |
|  | *eif-1* | 28.89 ± 0.5 | 31 | 64/64 | 21.57 ±0.4 | 23 | 49/53 | 34 | <.0001 |
|  | *eif-1* | 28.35 ± 0.4 | 30 | 42/45 | 22.90 ± 0.5 | 26 | 62/65 | 24 | <.0001 |
|  | *eif-1* | 26.90 ± 0.5 | 30 | 55/59 | 21.46 ± 0.3 | 23 | 55/60 | 25 | <.0001 |
|  | *eif-1* | 30.68 ± 0.3 | 32 | 72/76 | 23.41 ± 0.5 | 26 | 65/68 | 31 | <.0001 |
|  | *eif-1* | 27.51± 0.9 | 28 | 53/58 | 20.40 ± 0.5 | 20 | 50/50 | 35 | <.0001 |
|  | *eif-1* | 24.92 ± 0.7 | 29 | 52/54 | 22.43 ± 0.4 | 25 | 49/50 | 11 | <.0001 |
|  | *eif-1A* | 29.04 ± 0.5 | 32 | 69/71 | 22.75 ± 0.4 | 25 | 57/58 | 28 | <.0001 |
|  | *eif-1A* | 35.40 ± 0.6 | 39 | 57/59 | 23.15 ± 0.4 | 25 | 47/50 | 53 | <.0001 |
| *skn-1(zu135)* | ** ife-2* | 23.19 ± 0.6 | 23 | 51/57 | 21.96 ± 0.3 | 24 | 56/62 | 6 | .001 |
|  | ** ife-2* | 22.12 ± 0.7 | 26 | 49/63 | 19.45 ± 0.4 | 20 | 55/62 | 14 | <.0001 |
|  | ** ife-2* | 18.15 ± 0.3 | 18 | 53/53 | 18.09 ± 0.3 | 20 | 57/60 | 0 | .739 |
|  | ** ifg-1* | 29.50 ± 0.7 | 33 | 66/71 | 22.23 ± 0.3 | 25 | 109/112 | 33 | <.0001 |
|  | **ifg-1* | 20.90 ± 0.6 | 24 | 60/78 | 18.40 ± 0.3 | 21 | 71/86 | 14 | <.0001 |
|  | ** ifg-1* | 28.28 ± 0.9 | 32 | 49/55 | 21.96 ± 0.3 | 24 | 56/62 | 29 | <.0001 |
|  | ** eif-1* | 20.00 ± 0.5 | 21 | 62/62 | 18.09 ± 0.3 | 20 | 57/60 | 11 | .0551 |
|  | ** eif-1* | 24.69 ± 0.5 | 27 | 39/39 | 20.25 ± 0.6 | 24 | 47/49 | 22 | <.0001 |
|  | ** eif-1* | 21.94 ± 0.6 | 25 | 51/51 | 20.14 ± 0.4 | 22 | 49/51 | 9 | <.0001 |
|  | ** eif-1* | 23.09 ± 0.9 | 27 | 55/58 | 17.76 ± 0.6 | 23 | 58/60 | 30 | <.0001 |
|  | ** eif-1A* | 21.03 ± 0.5 | 24 | 61/62 | 18.06 ± 0.6 | 21 | 48/51 | 16 | .0086 |
|  | ** eif-1A* | 22.42 ± 0.6 | 23 | 45/45 | 17.82 ± 0.5 | 21 | 49/49 | 26 | <.0001 |
| *skn-1(zu67)* | *ife-2* | 17.86 ± 0.7 | 20 | 57/57 | 19.17 ± 0.7 | 23 | 56/60 | -7 | .1647 |
|  | *ife-2* | 18.70 ± 0.6 | 22 | 55/69 | 17.49 ± 0.5 | 22 | 55/62 | 7 | .0021 |
|  | *ifg-1* | 27.62 ± 0.9 | 32 | 50/56 | 19.17 ± 0.7 | 23 | 56/60 | 44 | <.0001 |
|  | *ifg-1* | 24.18 ± 0.7 | 29 | 51/55 | 17.49 ± 0.5 | 22 | 55/62 | 38 | <.0001 |
|  | *eif-1* | 23.64 ± 0.8 | 28 | 39/47 | 17.49 ± 0.5 | 22 | 55/62 | 35 | <.0001 |
|  | *eif-1* | 20.83 ± 0.8 | 24 | 45/52 | 16.69 ± 0.6 | 16 | 55/56 | 25 | .0013 |
|  | **eif-1* | 18.44 ± 0.7 | 17 | 48/50 | 17.81 ± 0.5 | 18 | 57/57 | 4 | .9303 |
|  | ** eif-1* | 19.24 ± 0.3 | 21 | 50/51 | 19.05 ± 0.3 | 21 | 42/46 | 1 | .05527 |
| *daf-16(mgDf47)* | *♦ ife-2* | 22.53 ± 0.5 | 25 | 57/63 | 22.52 ± 0.4 | 24 | 56/58 | 0 | .1881 |
|  | *♦ ife-2* | 17.46 ± 0.5 | 19 | 68/68 | 17.02 ± 0.4 | 19 | 57/58 | 3 | .2604 |
|  | *♦ ifg-1* | 23.63 ± 0.8 | 27 | 51/53 | 22.52 ± 0.4 | 24 | 56/58 | 5 | <.0001 |
|  | *♦ ifg-1* | 21.23 ± 0.7 | 26 | 63/64 | 17.02 ± 0.4 | 19 | 57/58 | 25 | <.0001 |
|  | ** ifg-1* | 26.53 ± 0.4 | 29 | 51/51 | 23.80 ± 0.3 | 25 | 55/55 | 11 | <.0001 |
|  | *♦ eif-1* | 18.63 ± 0.6 | 22 | 60/62 | 17.02 ± 0.4 | 19 | 57/58 | 11 | .0038 |
|  | *♦ eif-1* | 19.76 ± 0.6 | 23 | 49/56 | 17.88 ± 0.4 | 19 | 60/60 | 11 | .0018 |
|  | ** eif-1* | 18.06 ± 0.3 | 20 | 48/49 | 17.14 ± 0.2 | 18 | 56/56 | 5 | .0003 |
|  | ** eif-1* | 25.32 ± 0.4 | 29 | 71/71 | 23.80 ± 0.3 | 25 | 55/55 | 6 | .0002 |
|  | ** eif-1* | 21.18 ± 0.3 | 23 | 51/51 | 18.71 ± 0.3 | 20 | 47/47 | 13 | <.0001 |
| *daf-16(mgDf47); skn-1(zu67)* | *♦ ifg-1* | 20.66 ± 0.8 | 26 | 56/57 | 18.02 ± 0.6 | 20 | 50/54 | 15 | <.0001 |
|  | *♦ ifg-1* | 15.59 ± 0.5 | 16 | 59/62 | 15.60 ± 0.4 | 16 | 51/56 | 0 | .5289 |
|  | *ifg-1* | 20.51 ± 0.5 | 23 | 39/48 | 17.18 ± 0.4 | 19 | 40/50 | 19 | <.0001 |
|  | *♦ eif-1* | 16.04 ± 0.6 | 19 | 50/52 | 15.60 ± 0.4 | 16 | 51/56 | 3 | .2298 |
|  | *♦ eif-1* | 17.53 ± 0.5 | 21 | 50/54 | 16.02 ± 0.4 | 18 | 55/55 | 9 | .1251 |
|  | **eif-1* | 15.17 ± 0.3 | 14 | 52/52 | 16.07 ± 0.4 | 18 | 55/55 | -6 | .0028 |
|  | *eif-1* | 17.17 ± 0.3 | 18 | 41/46 | 17.39 ± 0.4 | 19 | 33/40 | -1 | .8322 |
| N2; +glc | *ife-2* | 19.01 ± 0.2 | 21 | 59/59 | 19.21 ± 0.3 | 20 | 56/56 | -1 | .6391 |
|  | *ife-2* | 20.73 ± 0.3 | 18 | 70/73 | 19.30 ± 0.2 | 20 | 71/71 | -7 | <.0001 |
|  | *ife-2* | 19.98 ± 0.2 | 22 | 59/59 | 22.49 ± 0.2 | 24 | 51/51 | -11 | <.0001 |
|  | *ifg-1* | 20.67 ± 0.3 | 23 | 58/58 | 19.21 ± 0.3 | 20 | 56/56 | -8 | <.0001 |
|  | *ifg-1* | 16.71 ± 0.2 | 23 | 51/52 | 19.30 ± 0.2 | 20 | 71/71 | -13 | <.0001 |
|  | *ifg-1* | 20.85 ± 0.4 | 23 | 54/54 | 22.49 ± 0.2 | 24 | 51/51 | -7 | .1214 |
|  | *eif-1* | 20.27 ± 0.4 | 23 | 67/67 | 19.21 ± 0.3 | 20 | 56/56 | -5 | .0004 |
|  | *eif-1* | 17.92 ± 0.2 | 19 | 60/60 | 19.30 ± 0.2 | 20 | 71/71 | -7 | <.0001 |
|  | *eif-1* | 21.69 ± 0.2 | 23 | 48/49 | 22.49 ± 0.2 | 24 | 51/51 | -4 | .0027 |
|  | *eif-1A* | 19.12 ± 0.3 | 20 | 64/64 | 22.49 ± 0.2 | 24 | 51/51 | -15 | <.0001 |

* Performed in parallel with an RNAi experiment done in N2; *♦* Performed in parallel with an RNAi experiment done in *skn-1(zu67).*
