# Supplementary material for: RNAi Screening Implicates a SKN-1–Dependent Transcriptional Response in Stress Resistance and Longevity Deriving from Translation Inhibition
Source: PLoS Genet. 2010 Aug 5;6(8):e1001048. doi: 10.1371/journal.pgen.1001048 (PMC2916858; doi:10.1371/journal.pgen.1001048)
Supplement: Table S6 — Strains used in this study, with references. (0.04 MB DOC) [file pgen.1001048.s009.doc]

Table S6. Strains used in this study.

| Number | Genetic background | Transgene | Array number | Reference |
| --- | --- | --- | --- | --- |
| LD1002 | N2 | Ex[*gcs-1p::GFP*] | 003 | [11] |
| LD1001 | N2 | Ex[*gcs-1p∆2::GFP*] | 004 | [11] |
| LD1000 | N2 | Ex[*gcs-1p∆2mut3::GFP*] | 005 | [11] |
| LD002 | N2 | Is[*SKN-1B/C::GFP*] | 007 | [11] |
| LD1008 | N2 | Ex[*SKN-1op::GFP*] | 009 | [17] |
| LD1171 | N2 | Is[*gcs-1p::GFP*] | 003 | This study |
| TJ356 | N2 | Is[DAF-16::GFP] |  | [28] |
|  | N2 | [*gst-4p::GFP*] |  | [62] |
|  | N2 | [*sod-3p::GFP*] |  | [63] |
| LD1173 | *skn-1(zu67)* | Is[*gcs-1p::GFP*] | 003 | This study |
| LD1175 | *skn-1(zu67)* | Is[*gcs-1p::GFP*] | 003 | This study |
| LD1260 | *daf-16(mgDf47)* | Ex[*gcs-1p::GFP*] | 003 | [17] |
|  | *sek-1(km4)* | Ex[*gcs-1p::GFP*] | 003 | [41] |
| EU1 | *skn-1(zu67)* | - | - | [59] |
| EU31 | *skn-1(zu135)* | - | - | [59] |
| LD1263 | *daf-16(mgDf47);skn-1(zu67)* | - | - | This study |
|  | *daf-16(mgDf47)* | - | - | [60] |
| KU4 | *sek-1(km4)* | - | - | [41] |
